# Germinal epimutation of Fragile Histidine Triad (FHIT) gene is associated with progression to acute and chronic adult T-cell leukemia diseases

**DOI:** 10.1186/s12943-021-01370-2

**Published:** 2021-06-06

**Authors:** Marcia Bellon, Izabela Bialuk, Veronica Galli, Xue-Tao Bai, Lourdes Farre, Achilea Bittencourt, Ambroise Marçais, Michael N. Petrus, Lee Ratner, Thomas A. Waldmann, Vahid Asnafi, Antoine Gessain, Masao Matsuoka, Genoveffa Franchini, Olivier Hermine, Toshiki Watanabe, Christophe Nicot

**Affiliations:** 1 Department of Pathology and Laboratory Medicine, University of Kansas Medical Center, Kansas City, KS 66160 USA; 2 Animal Models and Retroviral Vaccines Section, Vaccine Branch, Center for Cancer Research, National Cancer Institute, National Institutes of Health, Bethesda, MD USA; 3 Comprehensive Cancer Center, Department of Health Sciences, Ohio State University, Columbus, OH USA; 4 Program in Molecular Mechanisms and Experimental Therapy in Oncology, IDIBELL, Hospitalet de Llobregat, Barcelona, Spain; 5 Department of Pathology, Prof. Edgard Santos Teaching Hospital, Federal University of Bahia, Salvador, Bahia Brazil; 6 Institut Necker-Enfants Malades, Institut National de la Santé et de la Recherche Médicale U1151, Laboratoire Onco-Hématologie, Paris, France; 7 Lymphoid Malignancies Branch, Center for Cancer Research, National Cancer Institute, National Institutes of Health, Bethesda, MD USA; 8 Division of Oncology, Department of Medicine, Washington University, St Louis, MO USA; 9 Université de Paris (Descartes), Institut Necker-Enfants Malades, Institut National de la Santé et de la Recherche Médicale (INSERM) U1151 Laboratoire Onco-Hematology, Paris, France; 10 Unité d’épidémiologie et de Physiopathologie des virus Oncogene, Institut Pasteur, 75015 Paris, France; 11 Centre National de la Recherche Scientifique (CNRS) UMR 3569, 75015 Paris, France; 12 Laboratory of Virus Control, Institute for Frontier Life and Medical Sciences, Kyoto University, Kyoto, Japan; 13 Department of Hematology, Rheumatology, and Infectious Disease, Graduate School of Medical Sciences, Faculty of Life Sciences, Kumamoto University, Kumamoto, Japan; 14 Department of Hematology/Oncology, IMSUT Hospital, The Institute of Medical Science, The University of Tokyo, Tokyo, Japan

**Keywords:** HTLV-1, FHIT, ATL, ATLL, TSP, Leukemia, Lymphoma, Epigenetic, Methylation, Tax, TSP/HAM, HAM, Cancer, Epimutation

## Abstract

**Background:**

Human T cell Leukemia virus type 1 (HTLV-I) is etiologically linked to adult T cell leukemia/lymphoma (ATL) and an inflammatory neurodegenerative disease called HTLV-I-associated myelopathy or tropical spastic paraparesis (HAM/TSP). The exact genetic or epigenetic events and/or environmental factors that influence the development of ATL, or HAM/TSP diseases are largely unknown. The tumor suppressor gene, Fragile Histidine Triad Diadenosine Triphosphatase (FHIT), is frequently lost in cancer through epigenetic modifications and/or deletion. FHIT is a tumor suppressor acting as genome caretaker by regulating cellular DNA repair. Indeed, FHIT loss leads to replicative stress and accumulation of double DNA strand breaks. Therefore, loss of FHIT expression plays a key role in cellular transformation.

**Methods:**

Here, we studied over 400 samples from HTLV-I-infected individuals with ATL, TSP/HAM, or asymptomatic carriers (AC) for FHIT loss and expression. We examined the epigenetic status of FHIT through methylation specific PCR and bisulfite sequencing; and correlated these results to FHIT expression in patient samples.

**Results:**

We found that epigenetic alteration of FHIT is specifically found in chronic and acute ATL but is absent in asymptomatic HTLV-I carriers and TSP/HAM patients’ samples. Furthermore, the extent of FHIT methylation in ATL patients was quantitatively comparable in virus-infected and virus non-infected cells. We also found that longitudinal HTLV-I carriers that progressed to smoldering ATL and descendants of ATL patients harbor FHIT methylation.

**Conclusions:**

These results suggest that germinal epigenetic mutation of FHIT represents a preexisting mark predisposing to the development of ATL diseases. These findings have important clinical implications as patients with acute ATL are rarely cured. Our study suggests an alternative strategy to the current “wait and see approach” in that early screening of HTLV-I-infected individuals for germinal epimutation of FHIT and early treatment may offer significant clinical benefits.

**Supplementary Information:**

The online version contains supplementary material available at 10.1186/s12943-021-01370-2.

## Background

The HTLV-I virus is etiologically linked to a neurodegenerative disease, HTLV-I-associated myelopathy or tropical spastic paraparesis (TSP/HAM), and adult T-cell leukemia/lymphoma (ATL), a fatal T-cell leukemia stemming from infection with the HTLV-I virus [1, 2]. The etiology and pathogenesis of ATL diseases are not clear but the long latency period of > 20–40 years suggests that ATL disease results from virus-induced accumulation of genomic alterations [3]. The HTLV-I virus encodes an oncogenic protein, Tax, that plays an essential role in T-cell transformation. Among its roles, the HTLV-I Tax oncoprotein provokes DNA replication fork stalling and collapse, generates DNA double strand breaks (DSB) in dividing cells, and impairs homologous recombination (HR) repair of DSBs creating an environment conducive for the accumulation of genetic alterations [4, 5]. However, the presence of the virus alone or Tax functions are insufficient for disease progression since the lifetime cumulative risk of developing ATL is less than 5%; and most infected individuals remain asymptomatic. Several reports have described familial ATL cases [6, 7] which prompted us to hypothesize that a germinal alteration may predispose HTLV-I-infected individuals to ATL disease progression.

Studies using different approaches, such as genome-wide sequencing and transcriptome and methylome analyses have revealed numerous cellular genes altered in ATL samples [8]. Risk factors for AC patients to progress to ATL include higher proviral loads (PVLs), older age, family history of ATL and oligoclonal or monoclonal expansion of clones [9, 10]. High plasma levels of soluble tumor necrosis factor receptor 2 (sTNFR2) have been found in acute ATL, and could be used for diagnostic purposes [11]. A group of driver mutations (PLCG1, PRKCB, CCR4, TP53, and NOTCH1) have also been found in clonally expanded asymptomatic carrier patient cells of Afro-Caribbean lineage with high proviral loads that went on to develop aggressive ATL [12]; while sub-clonal mutations in the NF-κB/NFAT pathway have been found in the same geographical region of patients that relapsed or progressed from an indolent to aggressive ATL [13]. The mutational and transcriptional landscape of ATL patients from North America (which are predominately of Caribbean origin) also displayed similar mutations with a higher epigenetic mutational rates in EP300 [14]. Additional epigenetic or histone modifiers were also mutated; likely making these patients more susceptible to epigenetic treatment with DNA methyltransferase inhibitors. In a study focusing on TSP/HAM patient samples that progressed to ATL disease, those with a dominant clone and an ATL-like mutational signature went on to develop ATL [15].

Among the genes reported to be altered in a genome-wide methylome analysis, we selected FHIT as a potential candidate for an ATL predisposition factor because of its essential role in preserving genome integrity and its frequent inactivation in human cancers. The FHIT gene is found at 3p14. 2, a region with enhanced replication stress. This location contains the FRA3B gene, the most common fragile site in the genome that is involved in cancer induced chromosome rearrangements [16]. Due to FHIT’s location, deletions within FHIT are common in cancer. FHIT has been described as a tumor suppressor gene and disruption of one or both FHIT alleles leaves cells susceptible to carcinogen induced transformation [17, 18]. Over 50% of cancers show loss of FHIT expression; and it is believed that this loss is one of the earliest initiation events in the establishment of cancers [19]. In fact, reduced FHIT expression has been suggested to drive initiation of the specific cancer mutational signature [20]. Several observations suggest that the cumulative effect of Tax expression and lack of FHIT expression may generate a permissive environment for cellular transformation. Similar to p53, FHIT is considered a genome caretaker – with FHIT expression preventing replication stress and opposing replication forks stalling and forks collapse, while preventing the accumulation of DSBs during DNA replication [21, 22]. FHIT also has pro-apoptotic properties, activating caspases − 3, − 8, and – 9 [23, 24]. Consequently, it has been shown that FHIT-deficient cells are prone to acquire cancer promoting mutations and are more easily transformed in the presence of DNA damaging agents [25]. Loss of FHIT expression; therefore, leads to a conducive environment in early neoplastic cells for subsequent mutations in known tumor suppressor or oncogenic genes. It could then be hypothesized that upon HTLV-I infection, T-cells without FHIT would then be highly favorable to the viral transformation properties of Tax. To date, no study has examined the level or integrity of the FHIT gene in HTLV-I associated diseases. While loss of FHIT expression can occur due to chromosome breaks, FHIT is also highly methylated in solid tumors, such as lung, breast, bladder, prostate, cervical, esophageal, and hepatocellular cancers [26]. In hematological malignancies, FHIT methylation has been seen in some cases of myelodysplastic syndrome (MDS), acute myeloid leukemia (AML), and acute lymphoblastic leukemia (ALL), and chronic myelogenous leukemia (CML) [27–29]. Given that FHIT is a strong tumor suppressor and considered an early hit in the development of cancer, we examined the status of FHIT in HTLV-I associated diseases. We found FHIT was highly methylated in ATL patient samples, compared to healthy donors or other HTLV-I infected samples. Methylation of the FHIT gene corresponded to loss of FHIT expression in ATL patient samples. Notably, we found FHIT was comparably methylated in infected and non-infected cells in ATL patient samples. This suggests that loss of FHIT may serve as a driver event during HTLV-I-mediated T-cell transformation.

## Methods

### Patients samples

This retrospective cross-sectional study was carried out using PBMCs collected and immediately frozen. All samples used here are part of previous studies, for which these samples were collected after informed consent and approved by appropriate institutional review boards. According to the revised Shimoyama classification [30], 124 patients had acute ATL, 44 had chronic ATL, 20 had smoldering ATL, and 10 had lymphoma ATL. In addition, 136 patients had TSP/HAM, 89 patients were asymptomatic carriers (ACs), and 39 patients were healthy donors (HDs) not infected with HTLV-1. All samples were taken from blood, with no tissue biopsy. HTLV-1 proviral load was quantified for the majority of samples. The PVLs for all sub-types of ATL, TSP, and ACs were re-assessed using a standard method to compare HTLV-I PVLs. This was necessary to eliminate any disparity between patient samples due to the wide range of geographical areas and therefore different methods of gDNA extraction, purification, and PVL determination. In addition, due to the long-term collection of patient samples over many years, a standard PVL method was required. Upon arrival in the lab, patient samples were reassessed for gDNA integrity, diluted to 0.0125 μg/μl, and sample integrity and normalization was determined by use of qRT-PCR of GAPDH expression. A standard curve of pcTax plasmid DNA was used to determine copy number by qRT-PCR for Tax levels using Taqman probes; and was mixed with HD gDNA to determine copy number per cell. To compare the PVLs from different sample collections, these single use aliquots of 100 copy Tax DNA were generated, stored at − 80, and run on every qRT-PCR plate. Tax expression was then amplified from the gDNA of each patient sample and compared to the single-use aliquot of Tax/HD DNA. PVLs are determined per 400 cells. Patient data is provided in Table 1. The MT4 cell line, an HTLV-I positive, transformed cell line established from a 50-year-old Japanese male with ATL [31], was used as control. MT4 cells were grown in RPMI with 10% FBS.

### DNA extraction, bisulfite genomic sequencing and analysis

Genomic DNA (gDNA) was extracted from samples using DNAzol (ThermoFisher), and treated with Proteinase K, according to the manufacturer’s instructions. gDNA was treated with bisulfite using the MethylCode Bisfulfite Conversion Kit (ThermoFisher) and equal amounts of bisulfite DNA were used in methylation specific PCR (MSPCR) reactions. Equal amounts of PCR product were loaded onto TBE gels and visualized with SYBR green I nucleic acid stain (Molecular Probes). Primers used are described in Suppl 2. Methylation status was determined for each patient according to MSPCR results. For reference and to determine methylation status, bisulfite DNA from an UM and M patient were mixed to create a gradient for PCR detection (Suppl 1A). In all cases were MSPCR result was ambiguous, bisulfite genomic sequencing (BGS) was performed. Briefly, bisulfite DNA was amplified using specific primers using nested PCR. PCR products were cut from agarose gels, DNA was purified using QIAquick Gel Extraction kits (Qiagen), and ligated into the pGEM-T Easy Vector kit (Promega). Ligations were transformed into DH5a bacterial strain and individual colonies were selected for sequencing based on standard blue/white colony selection methods for miniprep DNA extraction and subsequent sequencing (example in Suppl 1B). Primers used are described in Table 2. For nail DNA extraction: clipped fingernail fragments were placed in an Eppendorf tube, washed in an acid buffer (pH 4) for 15 min, rinsed with TBE, and then incubated in a 1 M NaOH solution for 4 h. The solution was removed, and fragments were washed twice with 500ul of TBE, pH 7.5 and incubated overnight at 65C in presence of proteinase K. Genomic DNA was then extracted by chloroform phase centrifugation and ethanol precipitation; and gDNA was treated with bisulfite (as stated above).

### mRNA extraction and amplification

PBMC were isolated by ficoll centrifugation and RNA were extracted by TRIzol (ThermoFisher) lysis reagent, treated with TURBO DNAse (ThermoFisher), and used for RNA-to-cDNA amplification (Applied Biosystems). RNA expression was determined using iTaq Universal SYBR Green Supermix (Biorad) or iTaq Universal Probes Supermix (Biorad) with the StepOnePlus real-time instrument (AppliedBiosystems). Primers are described in Supplemental 2.

### Statistics

Odd risk ratios and chi-square statistics were determined for different HTLV-I diseases (Acute, chronic, smoldering, and lymphoma ATL) against HD, AC, or TSP patients for FHIT methylation. Odd risk ratios and their corresponding *p*-values and confidence intervals are provided in Supplemental 3. Chi-square results were determined using X^2^ (degrees of freedom, *N* = sample size) = chi-square statistic value, *p* = *p* value). One-way ANOVA statistics were determined as needed and were performed using the Social Science Statistical calculator using the One-Way ANOVA Calculator, including turkey HSD. For expression data, significance was determined using a two-tailed T-test for two independent means with the formula t((N_1_–1) + (N_2_–11)) = the t statistic, with appropriate *p*-values. Pearson’s correlation coefficient and corresponding *p*-values were performed using the Social Science Statistical calculator.

### CD25 and CD19 cell sorting and FACS analysis

Cell sorting was performed by magnetic bead separation or cell sorting by FACS. For magnetic bead sorting, PBMCs were washed in PBS containing 2% FBS and incubated with pre-washed Dynabeads anti-CD25 (Fig. 3) or anti-CD19 pan B (Fig. 4) magnetic beads (Invitrogen) for 30 min at 4C. The positive selection fraction was isolated by placing the tube for 2 min in a magnet; and cells were lysed in DNAzol for DNA extraction and/or RNAzol for RNA extraction. For isolation of the negative fraction, cells were incubated for a second round with magnetic beads to remove residual CD25+ or CD19+ cells. The suspension was placed in a magnet and unbound cells were lysed in DNAzol for DNA extraction. For cell sorting by FACS, cells were sorted on a 20-parameter FACSAria (BD) instrument and FACSDiVa software was used for analysis. Purity was confirmed by one or both methods: an aliquot was used for FACS analyses with incubation with one of the following fluorescently labeled, anti-human monoclonal antibodies: PerCP Cy 5.5-CD4 (# 552838), APC-CD19 (HIB19/#555,415), Alexa 700-CD3 (#561805), PE-CD25 (#555432) and Aqua Blue LIVE/DEAD Fixable Dead Cell Stain (Invitrogen) for FACS analyses and/or qRT-PCR for Tax expression to determine HTLV-I positive cell sorting.

## Results

**Fig. 1:**
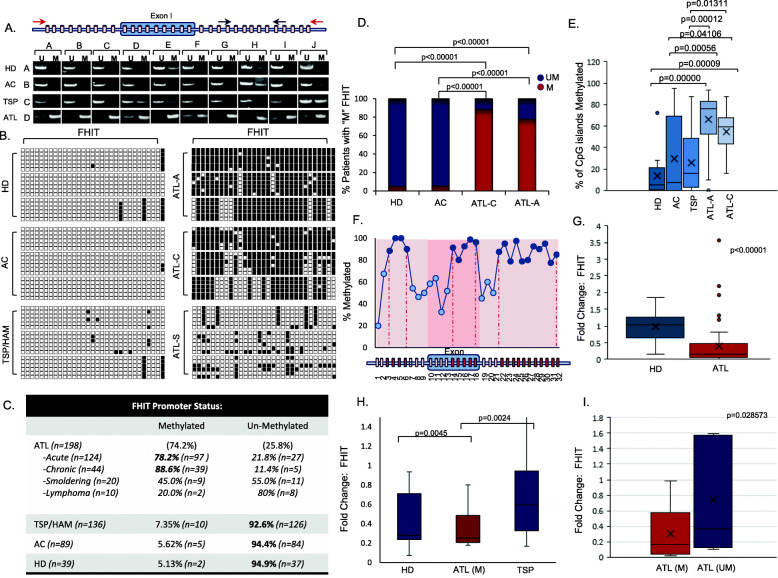
FHIT is highly methylated in ATL but not TSP patient samples and correlates with decreased FHIT expression. **a** Red and black arrows represent the amplicon for BGS and MSPCR, respectively of individual CpG islands (blocks). A representation of MSPCR for HDs (*n* = 10), ACs (*n* = 10), TSP/HAM (*n* = 10), and ATL (*n* = 10) samples are shown. Disease type is marked in columns A-D; while individual patients are marked in rows A-J. MSPCR amplifying the unmethylated (U) or methylated (M) FHIT gene product are shown; individual patients are noted by brackets. **b** Representation of BGS of HDs, ACs, TSP, and ATL patients (ATL-A: acute ATL, ATL-C, chronic ATL, and ATL-S, smoldering ATL). U vs M alleles are noted by white and black boxes, respectively. Horizontal rows represent sequencing of a single colony. **c** FHIT methylation in ATL (*n* = 198), ATL subtypes, TSP (*n* = 136), AC (*n* = 89), and HD (*n* = 39) from MSPCR. **d** Statistical analysis of FHIT methylation in HD, AC, and ATL samples. *p*-values are determined by Chi-Square test. **e** Representation of the overall percentage of M CpG islands (*n* = 32) in FHIT for HD (*n* = 12), AC (*n* = 10), TSP (*n* = 21), and ATL samples (chronic *n* = 46, acute *n* = 25). Results represent the average percentage of CpG islands in the analyzed portion of FHIT from BGS. The ANOVA f-ratio value is 16.67206, with a *p*-value of < .00001. Individual post hoc Tukey’s HSD *p*-values are noted when statistically significant between groups. **f** Distinct regions of the FHIT gene are methylated in ATL samples. The results are scored from the percentage of FHIT gene methylation at an individual CpG island from an average of approximately 20 acute ATL patients. **g** Quantitative real-time PCR performed on HDs (*n* = 38) and mostly acute ATL samples (*n* = 67) for FHIT gene expression. **h** Quantitative real-time PCR for FHIT gene expression in HDs (*n* = 15), methylated ATL (ATL-M; *n* = 16), and TSP/HAM (TSP; *n* = 14) samples. **i** Quantitative real-time PCR for FHIT gene expression in unmethylated (*n* = 7) vs methylated (*n* = 20) ATL patients. For (**g**-**i**), results were normalized to GAPDH expression and represent a fold change from a HD considered as “1”. *p*-values were calculated using a two-tailed, unequal variance T-test. FHIT CpG status was determined by MSPCR

An international collaborative effort was organized to collect over 400 samples from HTLV-I-infected individuals. To ensure genetic diversity of the tested population, samples originated from Asia, Africa, Europe, South and North America. We performed a comprehensive analysis on FHIT gene methylation and expression in uncultured peripheral blood mononuclear cells (PBMC) isolated from healthy volunteer donors (HD), HTLV-I infected asymptomatic carriers (AC), and HTLV-I infected individuals diagnosed with tropical spastic paraparesis/HTLV- I-associated myelopathy patients (TSP/HAM) and adult T-cell leukemia (ATL). Extracted genomic DNA was subject to bisulfite treatment and amplified using methylation-specific polymerase chain reaction (MSPCR) primers located in intron1 of the FHIT gene (Fig. 1a). Our results indicated that FHIT is unmethylated in normal healthy donors as well as in HTLV-I infected asymptomatic individuals and HTLV-I infected patients with TSP/HAM disease (Fig. 1a). In contrast, FHIT was strongly methylated in the majority of samples isolated from HTLV-I ATL patients (Fig. 1a). These data suggest that epigenetic modification of FHIT is characteristic of HTLV-I infected individuals with ATL disease. To further validate and confirm specificity, we performed bisulfite genomic sequencing (BGS) amplification of a region encompassing the 5′-end of the FHIT gene (Fig. 1b). The amplification product was cloned and for each sample five individual clones were sequenced. Sub-classification of ATL disease into acute, chronic, and smoldering subtypes demonstrated strong FHIT gene methylation in acute and chronic ATL patient samples, and moderate FHIT gene methylation in smoldering ATL patient samples (Fig. 1b). Representative data from these patients is presented (Fig. 1b). These data confirmed that positive FHIT gene methylation density results obtained using our MSPCR protocol are specific, accurate and correctly represent methylated CpG islands in the FHIT gene.

Having validated our MSPCR protocol, we then performed a large-scale analysis consisting of HD (*n* = 39), AC (*n* = 89), TSP/HAM (*n* = 136) and ATL (*n* = 198) patient samples. To ensure genetic background diversity and worldwide representation in our analysis, we obtained patient samples that originated from South America, Japan, Africa, Caribbean islands, Europe, and the United Sates (Table 1 and Suppl 4). Overall, our study demonstrated that a very high percentage of ATL patient samples (74%) presented with FHIT methylation. In contrast, only 7.35% for TSP, 5.6% for asymptomatic carriers, and 5.1% of healthy donor samples displayed FHIT methylation (Fig. 1c). The reasons underlying disease progression and whether a patient develops TSP/HAM and/or ATL are largely unknown. Our results suggest that methylated FHIT may play a major role in the pathogenesis of ATL and for the first time, clearly represents an epigenetic signature that differentiates TSP/HAM (7.35%) from acute and chronic ATL diseases. Among patients with ATL disease acute (74.2%) and chronic (88.6%) ATL patient samples were overwhelmingly represented by FHIT gene methylation, whereas 45 and 20% of smoldering and lymphoma-type ATL patient samples were found to have methylated FHIT. Importantly, all the ATL lymphoma samples analyzed were collected from PBMC where the presence of ATL cells is less than 5%. When compared to other viral lymphomas associated with infection by KSHV, HCV or EBV, ATL lymphoma patients presented with a higher proportion of methylated FHIT (Suppl 5); however, the limited number of samples precludes this from being statistically significant and will need to be expanded upon in future studies. Graphical representation and statistical analyses established that FHIT gene methylation in acute, chronic, and smoldering ATL subtypes, was statistically significant when compared to HD or asymptomatic samples (Fig. 1d). BSG analysis allowed us to examine approximately 32 individual CpG islands in the 5′-end of the FHIT gene in ATL patient samples. For most ATL patient samples, individual CpG islands were almost entirely methylated (Fig. 1e). However, when we randomly selected ATL patients for sequence analysis, we found that FHIT gene methylation displayed three peaks with very strong CpG island methylation that corresponded to regions within and just outside exon1 (Fig. 1f). A small drop in CpG island methylation was found outside exon 1 in the FHIT gene. It is possible that certain areas within the promoter and first exon of FHIT have a greater impact on FHIT gene expression. Further analysis will be required to determine if distinct areas of methylation are significant for FHIT expression in ATL disease.

The FHIT gene encompasses the FRA3B locus, a common fragile site deleted in cancer. In addition to methylation, FHIT inactivation occurs due to loss of heterozygosity, with a high frequency of deletions in exon 5, which harbors the FRA3B fragile site, and exon 8, which encompasses the histidine triad [32]. We analyzed ATL samples with unmethylated or weak methylated FHIT and found only 1 patient with a deletion of exon 5, and none with deletions in exon 8, demonstrating that the majority of unmethylated ATL patients’ sample have an intact FHIT gene, with no loss of FHIT integrity in these regions (Suppl 6). Examination of FHIT gene expression demonstrated significant decreases in ATL patients compared to HDs (Fig. 1g). HDs from different continents were included, to better match HDs to geographically different areas of ATL patient samples. Several outliers were noted with elevated expression of the FHIT gene. However, because matched DNA could not be obtained for all HD and ATL patient samples, an analysis against FHIT methylation could not be made. We, therefore, examined FHIT expression with matched gDNA samples for methylation analysis in HD, TSP, and ATL samples. FHIT gene methylation strongly correlated with lower FHIT mRNA expression compared to TSP patient and HD samples (Fig. 1h); and ATL patient samples with an unmethylated FHIT gene expressed lower levels compared to methylated ATL patient samples (Fig. 1i). Overall, these results clearly demonstrate that the FHIT gene is methylated in the majority of ATL patient samples, which correlates with decreased FHIT expression in ATL patient samples.

**Fig. 2:**
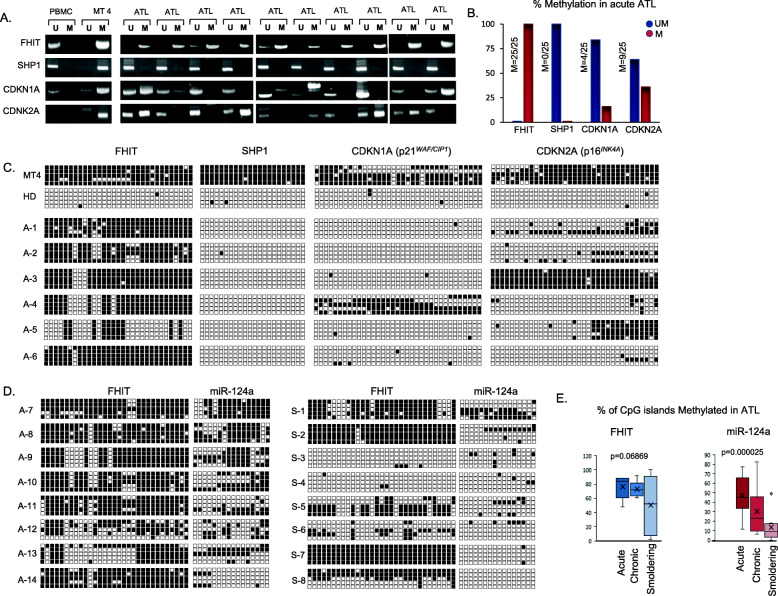
FHIT is preferentially methylated compared to known epigenetically regulated tumor suppressors (SHP1, CDKN1A, and CDKN2A) in ATL patients’ samples. **a** Representation of global MSPCR methylation patterns in a healthy, non-HTLV-I infected, PBMC, an HTLV-I (+) cell line, and identical acute ATL (*n* = 10) patients’ samples for FHIT, SHP1, CDKN1A, and CDKN2A. MSPCR bands amplifying the unmethylated (U) or methylated (M) product are represented. **b** The FHIT gene is methylated in acute ATL patients’ samples (*n* = 25) compared to SHP1, CDKN1A, and CDKN2A. The overall percentage of unmethylated (blue) or methylated (red) FHIT, SHP1, CDKN1A, and CDKN2A MSPCR products in identical acute ATL patients’ samples is graphed. **c** BSG analysis of individual CpG islands in the FHIT, SHP1, CDKN1A, and CDKN2A genes of corresponding ATL patients. BSG was used to determine the methylation pattern of MT4 (an HTLV-I+ cell line), a HD, and six identical acute ATL patients’ samples (A-1 through A-6). Unmethylated and methylated alleles are noted by white and black boxes, respectively; and each horizontal line represents a single amplified clone. **d** The FHIT gene does not show variations in methylation in different clinical subtypes of ATL. BSG sequencing was used to identify the global BSG methylation pattern of FHIT and miR-124a in eight identical acute ATL patients’ samples (A-7 through A-14), and eight identical smoldering ATL patients’ samples (S23-S30). **e** Graphical representation of the overall percentage of methylated (M) CpG islands (*n* = 31) in the FHIT gene and miR-124 microRNA (*n* = 16) in acute (ATL-A), chronic (ATL-C), and smoldering (ATL-S) samples from eight identical ATL patients. Patients are graphed from zero CpG islands methylated (0%) to complete CpG islands methylated (100%) in the analyzed portion of the FHIT and miR-124a genes. The values plotted represent an average value from sequencing of several BGSs. *p*-values were calculated by one-way ANOVA statistical test between the three groups

Increased genome methylation is frequently observed in cancer cells and genome wide methylation analyses of ATL patients’ samples has suggested that ATL tumor cells present with a CpG island methylator phenotype (CIMP) with increasing methylation in advanced ATL diseases [33]. On the other hand, evidence of familial ATL disease suggest the possible existence of a genetic predisposition marker [6, 7]. FHIT has been reported to be frequently methylated in tumor cells of various human cancers. We wanted to determine whether FHIT methylation occurs in ATL cells only because of ATL disease progression or if FHIT methylation is a preexisting germinal trait that may predispose some infected individuals to develop ATL diseases. We first examined several tumor suppressor genes that are known to be methylated specifically in ATL cells [34, 35]. MSPCR of FHIT, SHP1, CDNK1A (p21WAF1/CIP1), and CDNK2A (p14ARF/p16INK4a) were carried out in the same ATL patient samples. As expected, the FHIT gene was methylated in all ATL patient samples, whereas CDKN1A and CDKN2A displayed much lower gene methylation (Fig. 2a and b). While SHP1 has been reported to be methylated in ATL patients [36], our analysis demonstrated that SHP1 was unmethylated in all ATL patient samples tested. The discrepancy in SHP1 gene methylation most likely derives from non-specific, saturating conditions previously used in the SHP1 methylation PCR reaction [36]. To confirm that our MSPCR results represented the methylation density of these genes, we also performed BSG and found strong FHIT gene methylation in ATL patients compared to CDKN1A, CDKN2A, and SHP1 (Fig. 2c). Next, we investigated if FHIT methylation was associated or not with ATL disease progression. Acute, chronic, and smoldering ATL patient subtypes all displayed FHIT methylation that was not statistically different between subtypes (Fig. 2d and e). However, methylation of the microRNA, miR-124a, previously shown to be methylated in ATL cells [37], demonstrated ATL subtype specificity, whereby acute type ATL had statistically significant higher miR-124a methylation when compared to smoldering type ATL (Fig. 2d and e).

**Fig. 3:**
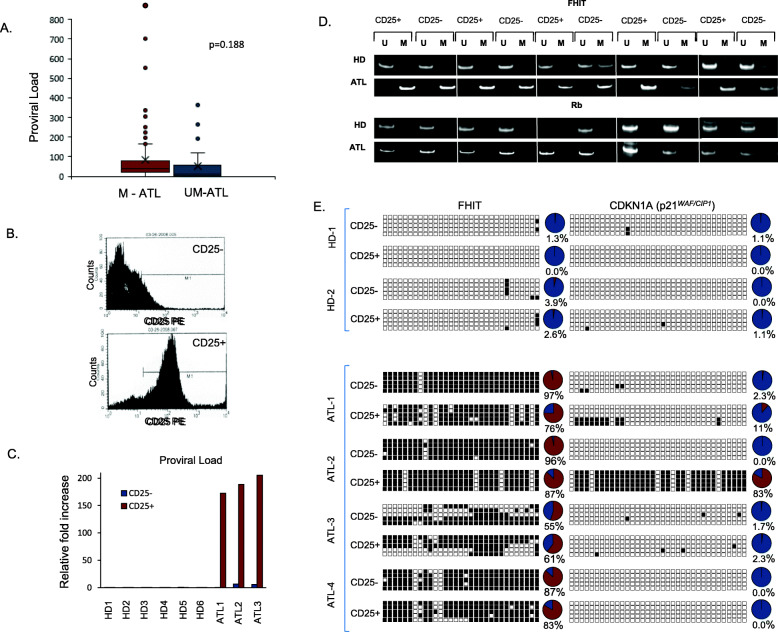
FHIT methylation is equally represented in both germinal and tumor cells and is independent from HTLV-I proviral load. **a** HTLV-I proviral load does not dictate FHIT gene methylation. Proviral loads were determined for acute and chronic ATL patients’ samples (*n* = 131 M and *n* = 32 UM) and compared to the epigenetic status of the FHIT gene as determined by MSPCR. *p*-values were obtained by two-tailed, unequal variance T-tests. **b** Effective sorting of ATL and HD PBMCs from patients into CD25- and CD25+ fractions by FACS analysis. 5 HDs and 5 ATL patients were sorted based on CD25 surface expression. **c** HTLV-I proviral load confirms effective CD25 sorting of HD and ATL patients’ samples. Proviral load was determined by quantitative PCR for gDNA isolated from CD25- and CD25+ samples. HTLV-I positive proviral loads were found in CD25+ fractions, compared to CD25-. Non-HTLV-I infected, HDs, served as negative controls. **d** FHIT CpG island methylation occurs in both tumorigenic (CD25+) and non-tumorigenic (CD25-) leukemic T-cells from ATL patients’ samples. Five non-HTLV-I infected HDs and five ATL patients’ samples (3 acute, 1 chronic, and 1 smoldering) were sorted into CD25- and CD25+ fractions. Individual MSPCR bands amplifying the unmethylated (U) or methylated (M) product are demonstrated. Methylation of CpG islands in the oncogene, Rb, was used as a control to demonstrate the specificity of the FHIT methylated PCR product. **e** Representation of global BSG methylation pattern of two HDs (HD-1 through HD-2), three acute ATL patients (ATL-1 through ATL-3) and one smoldering ATL (ATL-4) patient’s sample. Patient cells were sorted into CD25- and CD25+ fractions, and gDNA was analyzed for FHIT and CDKN1A gene methylation. Unmethylated and methylated alleles are noted by white and black boxes, respectively. Circle graphs representing the percentage of methylated CpG islands in the FHIT and CDKN1A genes are shown

ATL disease progression is associated with the expansion of tumor cells through cellular replication resulting in higher proviral loads [38]. In turn, one may suppose that increased methylation of FHIT may simply reflect the fact of higher tumor cells in the samples collected. However, when we examined the correlation between FHIT gene methylation status and HTLV-I proviral loads in acute and chronic ATL patient samples, we found no correlation (Fig. 3a). Consistent with these results, we found approximately 20% of ATL lymphoma patients had methylated FHIT. Since the percentage of circulating ATL cells is very low in the lymphoma type (less than 5%), these results strongly suggest that non-infected PBMC from lymphoma-type ATL carry a methylated FHIT gene. Altogether, our results suggest that FHIT gene methylation observed in ATL samples is not the consequence of CIMP and support the possibility of a preexisting germinal epimutation.

**Fig. 4:**
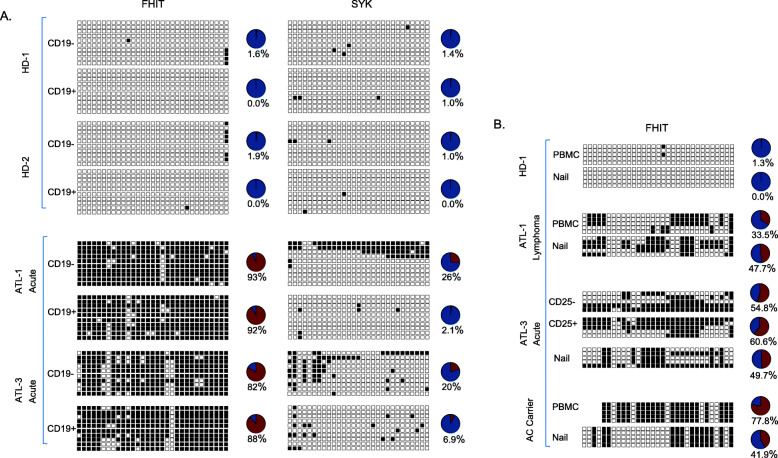
FHIT gene methylation is present in non-leukemic/non-T-cells in ATL patients’ samples. **a** FHIT gene methylation occurs in both tumorigenic (CD19-) and non-tumorigenic, B-cells (CD19+) in ATL patients’ samples. A representation of the global BSG methylation pattern of two HDs (HD-1 and HD-2) and two acute ATL patients (ATL-1 and ATL-3). Patient cells were sorted into CD19- and CD19+ fractions, and gDNA was analyzed for FHIT and SYK gene methylation. Unmethylated and methylated alleles are noted by white and black boxes, respectively. Circle graphs representing the percentage of methylated CpG islands in the FHIT and SYK genes are shown. **b** The FHIT gene is methylated in total PBMCs and cells from non-hematologic origin (nails) from the same individuals in ATL patients’ samples. A representation of the global BSG methylation pattern from total PBMCs and nails derived from the same HD (HD-1), lymphoma ATL (ATL-1), an acute ATL (ATL-3; same patient and data used for CD25+/in Fig. 3), and an asymptomatic/HTLV-I positive patient from an ATL+ family (see Fig. 5). gDNA corresponding to the same patient from PBMCs (tumorigenic) and nails were analyzed for FHIT gene methylation. For the acute ATL patient, PBMCs were further sorted into CD25- and CD25+ fractions. Unmethylated and methylated alleles are noted by white and black boxes, respectively. Circle graphs representing the percentage of methylated CpG islands in the FHIT and SYK genes are shown

To demonstrate this hypothesis, we decided to investigate the methylation status of FHIT in ATL patients’ cells not infected with HTLV-I. Circulating ATL cells are phenotypically characterized by markers CD3+/CD4+/CD25+; and CD3+/CD4+/CD25- cells are generally HTLV-I negative [39, 40]. Three acute, 1 chronic, and 1 smoldering ATL and five HD patient’s samples were used for CD4+/CD25- and CD4+/CD25+ cell sorting by FACS. The fraction’s purity was confirmed by using an aliquot for staining with anti- PE-CD25 conjugated antibody and FACS analyses (Fig. 3b). Purity of our fractionation was further confirmed by real time quantitative PCR of gDNA extracted from each fraction (Fig. 3c). As expected, amplification of the HTLV-I provirus sequence was detected in the CD25+ fraction of ATL samples (Fig. 3c). MSPCR results revealed the FHIT gene to be strongly and specifically methylated in both CD25- and CD25+ fractions in ATL samples but not in HDs (Fig. 3d). For these studies, the Rb gene was used as a control and expectedly the Rb gene was unmethylated in all fractions for both ATL and HDs (Fig. 3d). Next, we examined CD25−/+ fractions for FHIT gene methylation by BSG. We found the FHIT gene to not only be strongly methylated in both fractions of ATL patient samples, but also that the amount of CpG islands that were methylated to be almost equal between the leukemic (CD25+) and non-leukemic (CD25-) cells (Fig. 3e). These results exclude possible contamination of the CD25- fraction with few ATL positive cells. In the event of CD25+ cell contamination, then a significant percentage of the CpG islands analyzed by BSG would have been unmethylated in ATL patients’ samples, and this is clearly not the case. To detect equal CpG island methylation between fractions would require an approximate 50% contamination level, which is not supported by the FACS or the real time PCR data. Furthermore, BSG analyses of the CDKN1A gene demonstrated methylation only in the CD25+ fraction. These results are consistent with proper cell sorting and with the fact that CDKN1A is known to be specifically methylated in ATL cells. All these results further support the notion that in ATL patients, methylation of FHIT is not restricted to HTLV-I virus infected transformed ATL cells but rather methylated FHIT is present as a germinal alteration. Since in vivo HTLV-I-associated clonal expansion of transformed cells is restricted to CD4+ T cells [41], we next sorted ATL and HD samples into CD19+ and CD19- fractions. Interestingly, we found a similar FHIT gene methylation pattern between CD19+ and CD19- cell fractions in ATL samples tested (Fig. 4a). This was not the case with SYK gene methylation, preferentially expressed in B cells, whereby the CD19- fraction had significantly higher methylation of the SYK gene compared to the CD19+ fraction [42]. Once again, the comparable amount of CpG islands methylated in CD19- and CD19+ cell fractions support a germinal origin of methylated FHIT. Finally, we extracted gDNA from clipped nails of two ATL patients and one HDs and performed BSG. Results demonstrated FHIT methylation only in ATL patients’ nail samples (Fig. 4b).

**Fig. 5:**
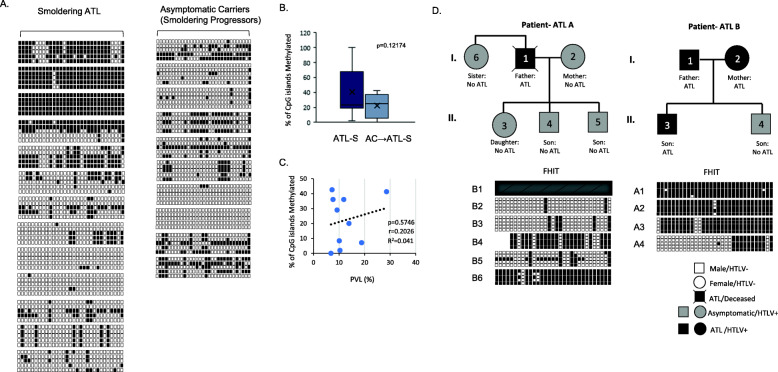
Longitudinal ACs that progress to Smoldering ATL and descendants of ATL patients harbor FHIT methylation. **a** FHIT CpG island methylation occurs in both overt smoldering ATL patients (*n* = 13) and ACs that progressed to smoldering ATL (*n* = 10). FHIT methylation was determined by BGS and unmethylated and methylated alleles are noted by white and black boxes, respectively. Due to the limited number of smoldering ATL patient samples, some patients are duplicated from previous figures (Figs. 1b and 2d). For progressors, FHIT methylation was determined at the time the patient was an AC, not when the patient developed ATL. **b** Graphical representation of the overall percentage of methylated (M) CpG islands (*n* = 31) in the FHIT gene in smoldering (ATL-S) samples (*n* = 13) and ACs that developed smoldering ATL (*n* = 10). Patients are graphed from zero CpG islands methylated (0%) to complete CpG islands methylated (100%). The values plotted represent an average value from sequencing of several BGSs. *p*-values were calculated by one-way ANOVA statistical test between the two groups. The f-ratio value is 2.61176. The *p*-value is 0.12174. **c** PVL does not correlate with the level of FHIT CpG methylation. PVLs were calculated as a % (as determined in a previous study [9]). PVL % was plotted against the % CpGs methylated. Pearson’s correlation coefficient and resulting *p*-value are indicated. **d** The primary descendants of two HTLV-I positive, ATL patients harbor methylation of the FHIT gene. The methylation status of the FHIT gene was determined for the immediate family members of two ATL patients’ samples. Patient samples were analyzed by BGS. Squares and circles denote male and female patients, respectively. White, grey, and black shapes represent non-HTLV-I infected, HTLV-I positive asymptomatic, and HTLV-I positive ATL, patients, respectively. For patient one (ATL A), the father, was deceased, and further analysis of the FHIT gene could not be performed. The BGS for patient B4 is identical to patient “AC carrier” in Fig. 4b

A longitudinal approach investigating asymptomatic carriers that progress to ATL is the best approach to establish FHIT methylation as a diagnostic marker for ATL disease. However, obtaining these longitudinal patient samples is extremely difficult due to the low percentage of asymptomatic carriers that develop ATL (2.5–5% lifetime risk) and the exceptionally long incubation period to acquire ATL disease (over 20 years) [9]. A recent study was performed examining the development of ATL disease in 1218 asymptomatic carriers from 2002 to 2008 that had enrolled in the Japanese Joint Study on Predisposing Factors of ATL Development (JSPFAD) [9]. During a median follow-up period of 1.0 year, 1.1% of the participants progressed to ATL disease (*n* = 14). We obtained gDNA from 10 patients that progressed to smoldering ATL. DNA was collected during the asymptomatic carrier stage of these patients and FHIT methylation analyzed and compared to samples from patients with smoldering ATL (Fig. 5a). We found no statistical difference in samples between smoldering ATL and asymptomatic carrier samples that will eventually progress to smoldering type ATL (ANOVA F-ratio = 2.6118, *p* = 0.12174, Fig. 5b). The proviral load did not correlate with the level of FHIT CpG methylation in these samples, nor age of the patient (Fig. 5c and data not shown). Our data suggests that asymptomatic carriers that develop smoldering ATL have levels of FHIT methylation comparable to patients with smoldering ATL disease and confirm predictive power of methylated FHIT for disease progression.

Finally, several studies have implied that ATL disease may cluster in families [6, 7]. A systemic review of literature suggests that different family members suffer from the same HTLV-1 disease and most families include two to four family members with disease [6]. Studies have shown that 19% of the variation in DNA methylation levels are contributed to additive genetic effects, largely due to genetic variants regulating methylation in a cis or trans manner [43–45]. Methylation can also be caused by non-additive genetic effects, such as parent-of-origin effects (POEs) where one inherited parental allele is responsible for the observed phenotypic effects, as is the case with genomic imprinting [43, 46]. If this is the case, FHIT methylation may be found in family members, exposing children of ATL affected family members to development of ATL. We obtained samples from two families, where at least one mother/father had confirmed ATL. We then examined FHIT methylation status in corresponding family members (Fig. 5d). In family A, the father died from ATL disease; however, we were able to obtain samples from a sibling, a wife, and three children that were all asymptomatic carriers for HTLV-I. Examination of FHIT methylation in all asymptomatic carriers of the family showed strong FHIT methylation in the sibling and son, with lower methylation in the 2nd son and daughter. This is remarkable, given that ACs have low FHIT methylation (5.62%). In the second family, the mother, father, and son all had ATL, while one son was an AC. Again, all family members demonstrated strong FHIT methylation, including the AC son. These results suggest that in ATL families, 1- FHIT methylation is higher in descendants with methylated FHIT (FHIT methylation = 100%) than in normal asymptomatic carriers (FHIT methylation = 5.62%), 2- children of ATL parents have strongly methylated FHIT regardless of whether they are asymptomatic carriers or have ATL, 3- FHIT methylation may be passed down from parents to children through additive or non-additive genetic effects. We were unable to follow the descendants and sibling long-term. However, our data suggests that they are at a higher risk of developing ATL in their lifetime due to the strong correlation between ATL disease and FHIT methylation presented in Fig. 1.

## Discussion

ATL diseases have a dismal prognosis with median survival times of 8.3 and 10.6 months and a 4-year overall survival rates of 11 and 16% for the acute and chronic ATL, respectively [47]. To date, a quick method to predict the risk of an individual infected with HTLV-I to developing ATL diseases has been largely undefined, effectively preventing early treatment. The backbone of ATL therapy is largely unchanged in the past several decades, with new treatment options including allogeneic hematopoietic stem-cell transplantation (allo-HSCT) or therapies such as mogamulizumab (anti-CC chemokine receptor 4 monoclonal antibody) or lenalidomide (immunomodulatory agent) being considered [48]. New treatment options vary considerably in different geographical regions, with countries such as Japan using drug therapies that are only available in the country (such as mogamulizumab and lenalidomide), while zidovudine (AZT) and interferon-alpha (INF-α) are used mostly in non-Japanese countries. Overall, asymptomatic carriers and smoldering ATL are often given the “wait and see approach”, indicating active monitoring, while some countries administer AZT/INF-α. Chronic and acute ATL treatment often involves AZT/INF-α, chemotherapy and/or allo-HSCT depending on the country [30, 48]. Reports suggest that if left untreated indolent type ATL will progress to the acute type. In fact, approximately half of the patients with chronic ATL progress to acute ATL within 18 months from diagnosis and die [49]. While multi-agent chemotherapies may worsen the prognosis of chronic ATL, when compared to watch and wait for active monitoring [49], early treatment with anti-retroviral therapy AZT/IFN produces long-term survival of chronic ATL patients [50]. This suggests that identifying HTLV-I asymptomatic individuals that have a high probability of developing ATL is essential in determining an accurate first line of therapy.

Our study identifies germinal FHIT methylation as a signature of ATL disease development that is present in more than 80% of HTLV-I-infected patients with acute or chronic ATL. A recent study has shown that the transcriptomic profile of North American ATL is distinct from Japanese ATL [14]. The fact that FHIT is methylated in ATL patients across all geographical regions demonstrates its usefulness as a broad prognostic marker for the development of ATL disease. The ability to identify and predict which HTLV-I infected asymptomatic patients have a significantly higher risk to develop ATL will allow initiation of treatment earlier with greater improvement on patients’ overall survival. A recent study of TSP/HAM patients from Japan suggests that approximately 3% of TSP/HAM patients develop ATL [15]. We found approximately 7% of TSP/HAM patients carry a methylated FHIT allele. While further longitudinal studies are required, it does open the possibility that FHIT methylation could also predict ATL development not only in ACs, but in other HTLV-I associated diseases. One limitation of our study is that the age distribution was unknown for all patient samples provided. Given that the development of ATL occurs in persons over 40 years of age, it is likely that most of the ATL patient samples used in this study were older. A study on age-related progression of FHIT methylation will need to be conducted in the future. However, FHIT methylation has not been shown to be dependent upon patient age or gender in any other cancers [51–53] suggesting this is also the case in ATL. It is also possible, though doubtful, that virus related factors produced by HTLV-I-infected cells are responsible for FHIT methylation. It remains highly unlikely due to several observations: 1- FHIT methylation was independent of proviral load, 2- non-infected cells (CD25- and CD19-) harbored methylated FHIT to the same extend as infected cells (arguing against the dilution effect), 3- gDNA extracted from nails was positive for FHIT methylation, and 4- the low number of FHIT methylated samples in asymptomatic carriers and TSP/HAM patients, who are infected with HTLV-I. We found approximately, 22% of acute and 11% of chronic ATL patient samples had unmethylated FHIT. Why some patients escape FHIT methylation is currently unknown. We do know that at least one UM ATL patient sample carried a deletion in exon 5. In our study, we only tested exons 4, 5, and 8 in a portion of the UM ATL patient samples. The FHIT gene comprises 10 exons, so it is possible that other portions of the FHIT gene were deleted or mutated creating early termination or frameshift. Additionally, FHIT is known to be affected by allelic loss, homozygous deletions, LOH, microsatellite instability, microRNAs, and lncRNAs [54, 55]. miR-143 and miR-29b have both been shown to regulate FHIT expression, and may have an impact in ATL disease [56, 57].

This is also the first study to address the expression of FHIT in HTLV-I infection and ATL disease. Studies have shown that loss of FHIT expression can direct a cell into a more genetically favorable cancer inducing phenotype. Given the long latency period between HTLV-I infection and the development of ATL disease, the loss of FHIT early on would be extremely beneficial. Besides it roles as a genome caretaker, FHIT has known roles in apoptosis, attenuating NF-κB and AKT/survivin signaling pathways, Ras/RhoGTPase, a repressor of the β-catenin pathway, interacts with the SUMO-1 conjugating enzyme, Ubc9, and plays a role in the cytoskeleton [26, 58–60]. These pathways are largely disrupted in ATL patient samples. While the Tax protein plays a role in some of these pathways, it is possible that loss of FHIT expression collaborates or enhances these actions of Tax or other HTLV-I proteins. Tax is highly immunogenic; therefore, HTLV-I infected cells carry minimal, but inducible levels of Tax expression [61]. Loss of FHIT expression would allow a favorable environment for HTLV-I infected cells to obtain cancer driver events, in the absence of high levels of Tax expression. Given that FHIT methylation was only seen in ATL patient samples, and not asymptomatic or TSP/HAM patient samples, again confirms its specific role in tumor initiation and/or maintenance. A recent study demonstrated that OR-2100, a novel decitabine prodrug, was successful in killing ATL cells through DNA hypomethylation, indicating that targeting DNA methylation could be effective in eliminating ATL cells [62]. It is also interesting to note that in our study less than 3% of healthy donors carry a methylated FHIT gene. This suggests that there is small amount of the population that may be susceptible to ATL disease if infected with HTLV-I. Whether these individuals are suspectable to other forms of cancer is unknown, but in highly endemic areas, screening for FHIT status and HTLV-I infection could allow for early identification of individuals that have a higher propensity to develop ATL. Also, FHIT methylation could be developed into a rapid test, which would be extremely beneficial in screening asymptomatic carriers. This would be vital for viral treatment options that could stop the cells from developing enough transformative events to develop ATL.

## Conclusion

In summary, we have identified the tumor suppressor gene, FHIT, as being highly disrupted in ATL disease. The majority of acute and chronic ATL patient’s cells carry methylated FHIT, which is present in tumorigenic and non-tumorigenic cells, and leads to loss of FHIT expression. This suggests that FHIT may be a predisposition marker for identification of HTLV-I infected individuals that will progress to ATL disease.

## Supplementary Information

**Additional file 1: Supplemental 1.** Development of MSPCR and BGS analysis for examination of FHIT methylation. (A) MSPCR was used to determine the FHIT methylation status in patient samples. A standard was derived by mixing bisulfite treated DNA from a patient with UM FHIT with bisulfite treated DNA from a patient with M FHIT. Ratios of 100:0, 75:25, 50:50, 25:75, and 0:100 were used in MSPCR for UM and M PCR, respectively. (B) Example of BSG sequencing of an UM and M patient sample. Arrows point to distinct CpG islands in the same DNA sequence of FHIT; demonstrating UM (C to T) or M (C remains) in bisulfite treated DNA. **Supplemental 2.** Primers used in FHIT study. Accuprime (Invitrogen) or Q-solution (Qiagen) was used for FHIT, miR-124a, CDKN1A, and CDKN2A PCRs. Two PCRS were carried out for FHIT, SHP1, SYK, CDKN1A, and CDKN2A. BGS PCR conditions were as follow: FHIT BGS: 95–30″, TD: 61–51–1′, 72–1′ (Touchdown). SHP1 BGS: 95–30″, 64–1′, 72–1′ (35-40c). SYK BGS: 95–30″, 49–30″, 72–30″ (40c). CDKN1A BGS: 95–30″, TD: 55–45-1′, 72–1′ (Touchdown). CDKN2A BGS: 95–30″, TD: 61–51–1′, 72–1′ (Touchdown). miR-124a BGS: 95–30′, 54–40″, 72–40″ (40c). **Supplemental 3.** Statistical analysis of HTLV-I diseases for FHIT methylation. Odd risk ratios and chi-square statistics were determined for different HTLV-I diseases (Acute, chronic, smoldering, and lymphoma ATL) against HD, AC, or TSP patients for FHIT methylation. Odd risk ratios and chi-square statistics were determined for different HTLV-I diseases (Acute, chronic, smoldering, and lymphoma ATL) against HD, AC, or TSP patients for FHIT methylation. Chi-square results were determined using X2 (degrees of freedom, *N* = sample size) = chi-square statistic value, *p* = *p* value). **Supplemental 4.** Geographical distribution of patient samples. Pie diagrams were used to illustrate the geographical distribution of ATL (acute, chronic, smoldering, and lymphoma type), TSP/HAM, and ACs. Continent of origin (Asia, Africa, North America (N.Amer.), South America (S.Amer.), and Europe) was determined from providers. If the continent of origin was not known at the time, the samples are marked as “unknown”. Acute ATL (*n* = 124), chronic ATL (*n* = 44), smoldering ATL (*n* = 20), lymphoma ATL (*n* = 20), TSP/HAM (*n* = 136), and asymptomatic (AC), HTLV-I carriers (*n* = 89). **Supplemental 5.** FHIT methylation in viral lymphomas. (A) Representation of BGS of lymphoma-type ATL patients. U vs M alleles are noted by white and black boxes, respectively. Horizontal rows represent sequencing of a single colony. (B) FHIT methylation status in viral lymphomas. HTLV-I, ATL-lymphoma (*n* = 10), Kaposi’s sarcoma herpesvirus (human herpesevirus-8) (KSHV) associated lymphoma (*n* = 13), Epstein-barr (EBV) associated Hodgkin’s lymphoma (HL) (*n* = 18), and hepatitis C virus (HCV) associated lymphomas (*n* = 15). Results were determined by MSPCR of FHIT. **Supplemental 6.** Deletional Analysis of the FHIT gene in ATL patients. (A) Chart representing UM (or very weak M) ATL patients for deletional analysis of the FHIT gene. One patient carried a deletion in exon 5. (B) Representation of the PCR bands for exon 4, 5, and 8. In some patients, exon 4 was also amplified. Genomic DNA was used to amplify different FHIT exons with the following primers: FHIT Exon 4: F-GACTAGGAATCAGAAATGAATAATTA, R-GCATGTCAGTCAGGTAACAGGTAAGC; FHIT Exon 5: F-GCTGTTTTATTGTCCACGTGGAAGCT, R-CTCAGCTATGGTAGTGAAAAGGTCAA; and FHIT Exon 8: F-GATGCACTGTCATTTCAAAGCACTGG, R-CATATCTCCATGCAAATATTTACTGTC.

**Additional file 2: Supplemental Table 1.**

## Data Availability

Data sharing is not applicable to this article as no datasets were generated or analyzed during the current study.

## Abbreviations

HTLV: Human T-cell leukemia virus
ATL: Adult t-cell leukemia/lymphoma
HAM/TSP: HTLV-I-associated myelopathy or tropical spastic paraparesis
FHIT: Fragile Histidine Triad Diadenosine Triphosphatase
FRA3B: Fragile Site, Aphidicolin Type, Common, Fra(3)(P14.2)
MSPCR: Methylation specific polymerase chain reaction
PBMC: Peripheral blood monocytes cells
HD: Healthy volunteer donors
AC: HTLV-I infected asymptomatic carriers
BGS: Bisulfite genomic sequencing
FACS: Fluorescence activated cell sorting
PCR: Polymerase chain reaction
HSD: Honestly significant difference
gDNA: Genomic DNA
M: Methylated
UM: Unmethylated
CIMP: CpG island methylator phenotype
CDKN1A: Cyclin Dependent Kinase Inhibitor 1A
CDKN2A: Cyclin Dependent Kinase Inhibitor 2A
SHP1: Protein Tyrosine Phosphatase Non-Receptor Type 6
SYK: Spleen Associated Tyrosine Kinase
DSB: DNA double strand breaks
HR: Homologous recombination
MDS: Myelodysplastic syndrome
AML: Acute myeloid leukemia
ALL: Acute lymphoblastic leukemia
allo-HSCT: Allogeneic hematopoietic stem-cell transplantation
CML: Chronic myelogenous leukemia
AZT: Azidothymidine
IFN: Interferon
NF-κB: Nuclear factor kappa-light-chain-enhancer of activated B cells
AKT: AKT Serine/Threonine Kinase 1
SUMO-1: Small Ubiquitin Like Modifier 1
Rho: Ras Homolog Family
Ubc9: Ubiquitin Conjugating Enzyme E2
HCV: Hepatitis C virus
EBV: Epstein-Barr virus
KSHV: Kaposi’s sarcoma herpesevirus (human herpesvirus-8)
JSPFAD: Joint Study on Predisposing Factors of ATL Development
POE: Parent-of-origin effects
miRNA: microRNA
lncRNA: Long non-coding RNA
LOH: Loss of heterozygosity
