# Supplementary material for: Germinal epimutation of Fragile Histidine Triad (FHIT) gene is associated with progression to acute and chronic adult T-cell leukemia diseases
Source: Mol Cancer. 2021 Jun 6;20:86. doi: 10.1186/s12943-021-01370-2 (PMC8183032; doi:10.1186/s12943-021-01370-2)
Supplement: Supplementary file 1 — Additional file 1: Supplemental 1. Development of MSPCR and BGS analysis for examination of FHIT methylation. (A) MSPCR was used to determine the FHIT methylation status in patient samples. A standard was derived by mixing bisulfite treated DNA from a patient with UM FHIT with bisulfite treated DNA from a patient with M FHIT. Ratios of 100:0, 75:25, 50:50, 25:75, and 0:100 were used in MSPCR for UM and M PCR, respectively. (B) Example of BSG sequencing of an UM and M patient sample. Arrows point to distinct CpG islands in the same DNA sequence of FHIT; demonstrating UM (C to T) or M (C remains) in bisulfite treated DNA. Supplemental 2. Primers used in FHIT study. Accuprime (Invitrogen) or Q-solution (Qiagen) was used for FHIT, miR-124a, CDKN1A, and CDKN2A PCRs. Two PCRS were carried out for FHIT, SHP1, SYK, CDKN1A, and CDKN2A. BGS PCR conditions were as follow: FHIT BGS: 95–30″, TD: 61–51–1′, 72–1′ (Touchdown). SHP1 BGS: 95–30″, 64–1′, 72–1′ (35-40c). SYK BGS: 95–30″, 49–30″, 72–30″ (40c). CDKN1A BGS: 95–30″, TD: 55–45-1′, 72–1′ (Touchdown). CDKN2A BGS: 95–30″, TD: 61–51–1′, 72–1′ (Touchdown). miR-124a BGS: 95–30′, 54–40″, 72–40″ (40c). Supplemental 3. Statistical analysis of HTLV-I diseases for FHIT methylation. Odd risk ratios and chi-square statistics were determined for different HTLV-I diseases (Acute, chronic, smoldering, and lymphoma ATL) against HD, AC, or TSP patients for FHIT methylation. Odd risk ratios and chi-square statistics were determined for different HTLV-I diseases (Acute, chronic, smoldering, and lymphoma ATL) against HD, AC, or TSP patients for FHIT methylation. Chi-square results were determined using X2 (degrees of freedom, N = sample size) = chi-square statistic value, p = p value). Supplemental 4. Geographical distribution of patient samples. Pie diagrams were used to illustrate the geographical distribution of ATL (acute, chronic, smoldering, and lymphoma type), TSP/HAM, and ACs. Continent of origin (Asia, Africa, North America (N.Amer.), [file 12943_2021_1370_MOESM1_ESM.pptx]

## Slide 1
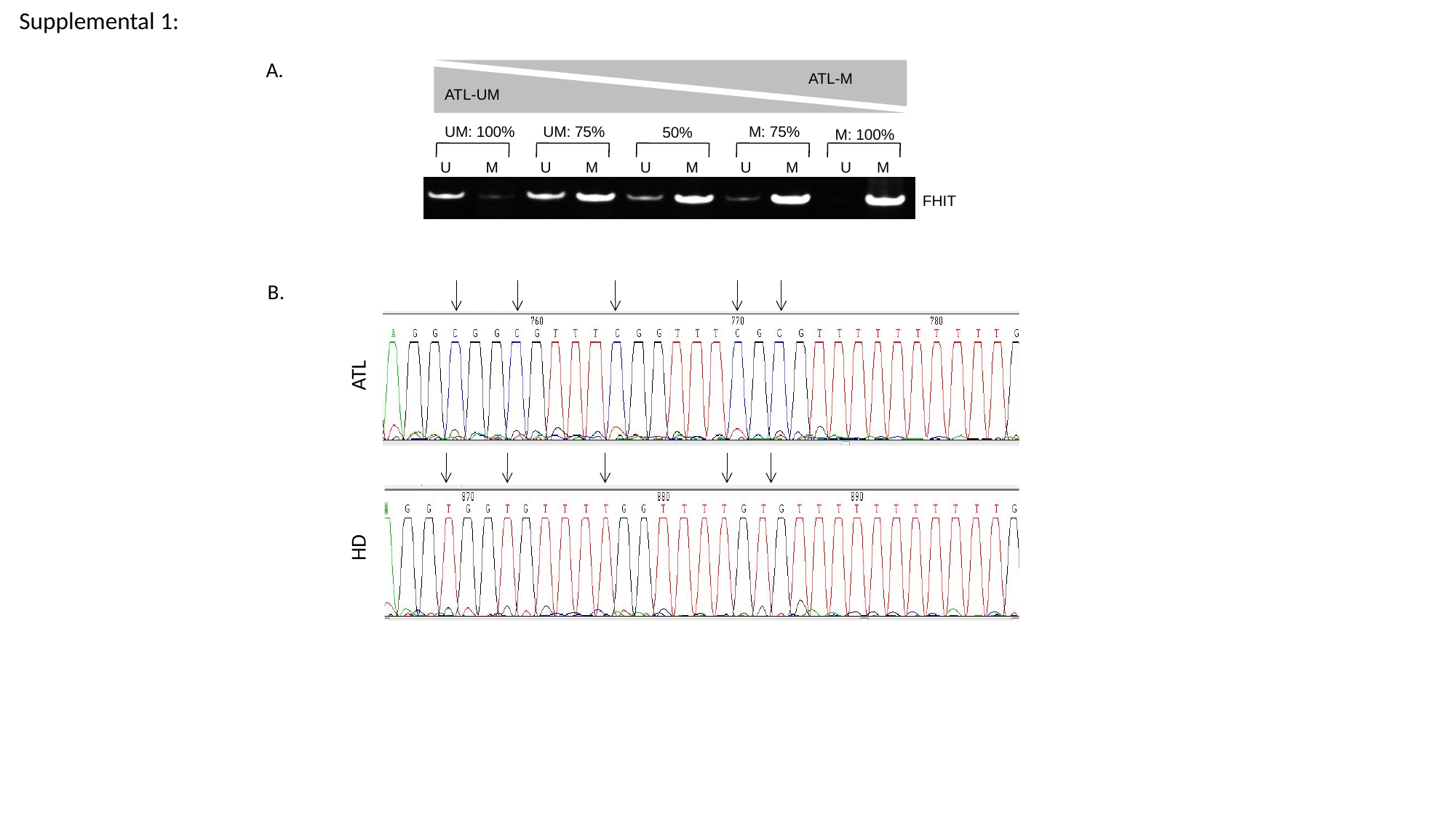

Supplemental 1:
A.
ATL-M
ATL-UM
UM: 75%
M: 75%
UM: 100%
50%
M: 100%
U
M
U
M
U
M
U
M
U
M
FHIT
B.
ATL
HD

## Slide 2
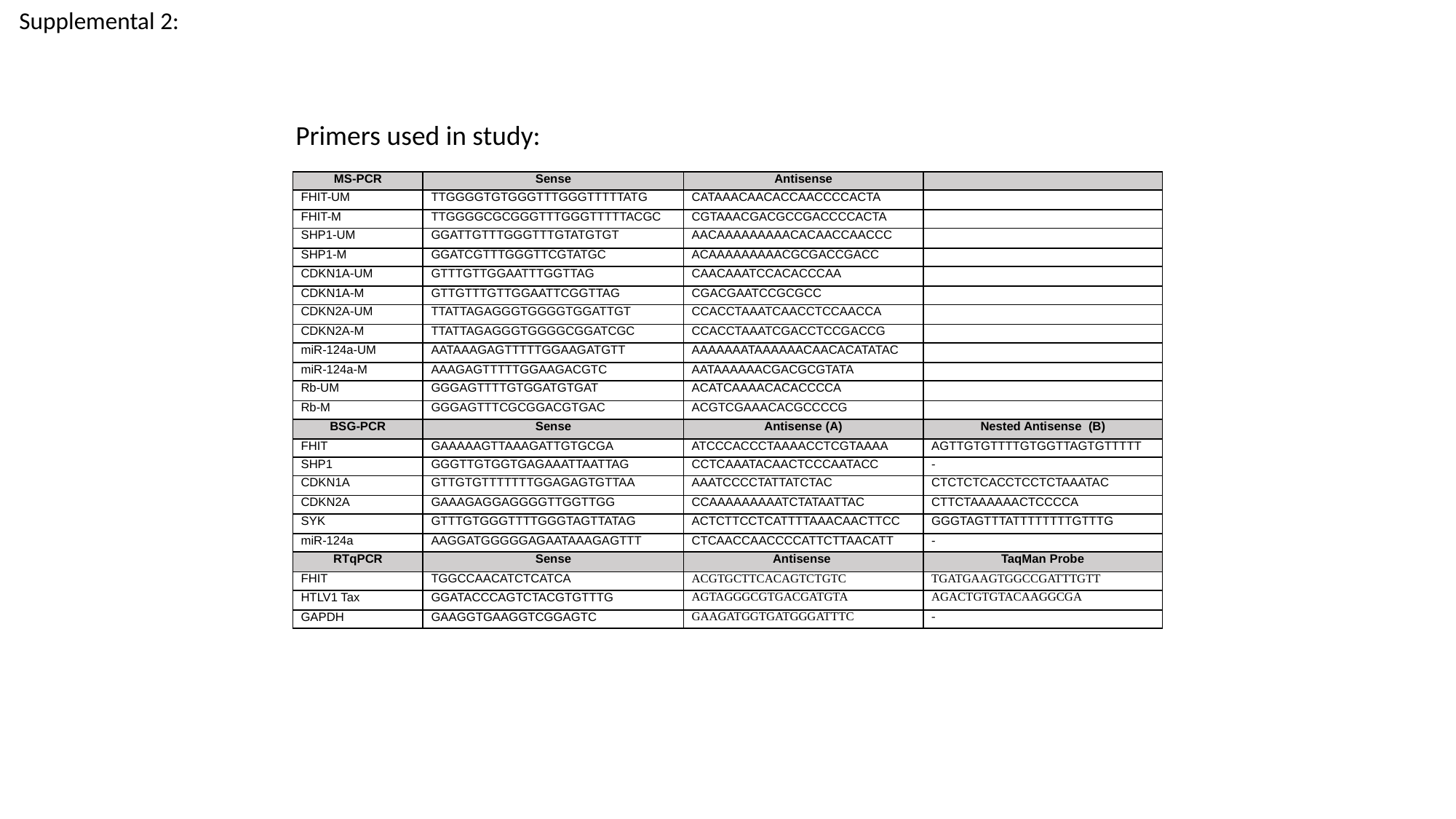

Supplemental 2:
Primers used in study:
| MS-PCR | Sense | Antisense | |
| --- | --- | --- | --- |
| FHIT-UM | TTGGGGTGTGGGTTTGGGTTTTTATG | CATAAACAACACCAACCCCACTA | |
| FHIT-M | TTGGGGCGCGGGTTTGGGTTTTTACGC | CGTAAACGACGCCGACCCCACTA | |
| SHP1-UM | GGATTGTTTGGGTTTGTATGTGT | AACAAAAAAAAACACAACCAACCC | |
| SHP1-M | GGATCGTTTGGGTTCGTATGC | ACAAAAAAAAACGCGACCGACC | |
| CDKN1A-UM | GTTTGTTGGAATTTGGTTAG | CAACAAATCCACACCCAA | |
| CDKN1A-M | GTTGTTTGTTGGAATTCGGTTAG | CGACGAATCCGCGCC | |
| CDKN2A-UM | TTATTAGAGGGTGGGGTGGATTGT | CCACCTAAATCAACCTCCAACCA | |
| CDKN2A-M | TTATTAGAGGGTGGGGCGGATCGC | CCACCTAAATCGACCTCCGACCG | |
| miR-124a-UM | AATAAAGAGTTTTTGGAAGATGTT | AAAAAAATAAAAAACAACACATATAC | |
| miR-124a-M | AAAGAGTTTTTGGAAGACGTC | AATAAAAAACGACGCGTATA | |
| Rb-UM | GGGAGTTTTGTGGATGTGAT | ACATCAAAACACACCCCA | |
| Rb-M | GGGAGTTTCGCGGACGTGAC | ACGTCGAAACACGCCCCG | |
| BSG-PCR | Sense | Antisense (A) | Nested Antisense (B) |
| FHIT | GAAAAAGTTAAAGATTGTGCGA | ATCCCACCCTAAAACCTCGTAAAA | AGTTGTGTTTTGTGGTTAGTGTTTTT |
| SHP1 | GGGTTGTGGTGAGAAATTAATTAG | CCTCAAATACAACTCCCAATACC | - |
| CDKN1A | GTTGTGTTTTTTTGGAGAGTGTTAA | AAATCCCCTATTATCTAC | CTCTCTCACCTCCTCTAAATAC |
| CDKN2A | GAAAGAGGAGGGGTTGGTTGG | CCAAAAAAAAATCTATAATTAC | CTTCTAAAAAACTCCCCA |
| SYK | GTTTGTGGGTTTTGGGTAGTTATAG | ACTCTTCCTCATTTTAAACAACTTCC | GGGTAGTTTATTTTTTTTGTTTG |
| miR-124a | AAGGATGGGGGAGAATAAAGAGTTT | CTCAACCAACCCCATTCTTAACATT | - |
| RTqPCR | Sense | Antisense | TaqMan Probe |
| FHIT | TGGCCAACATCTCATCA | ACGTGCTTCACAGTCTGTC | TGATGAAGTGGCCGATTTGTT |
| HTLV1 Tax | GGATACCCAGTCTACGTGTTTG | AGTAGGGCGTGACGATGTA | AGACTGTGTACAAGGCGA |
| GAPDH | GAAGGTGAAGGTCGGAGTC | GAAGATGGTGATGGGATTTC | - |

## Slide 3
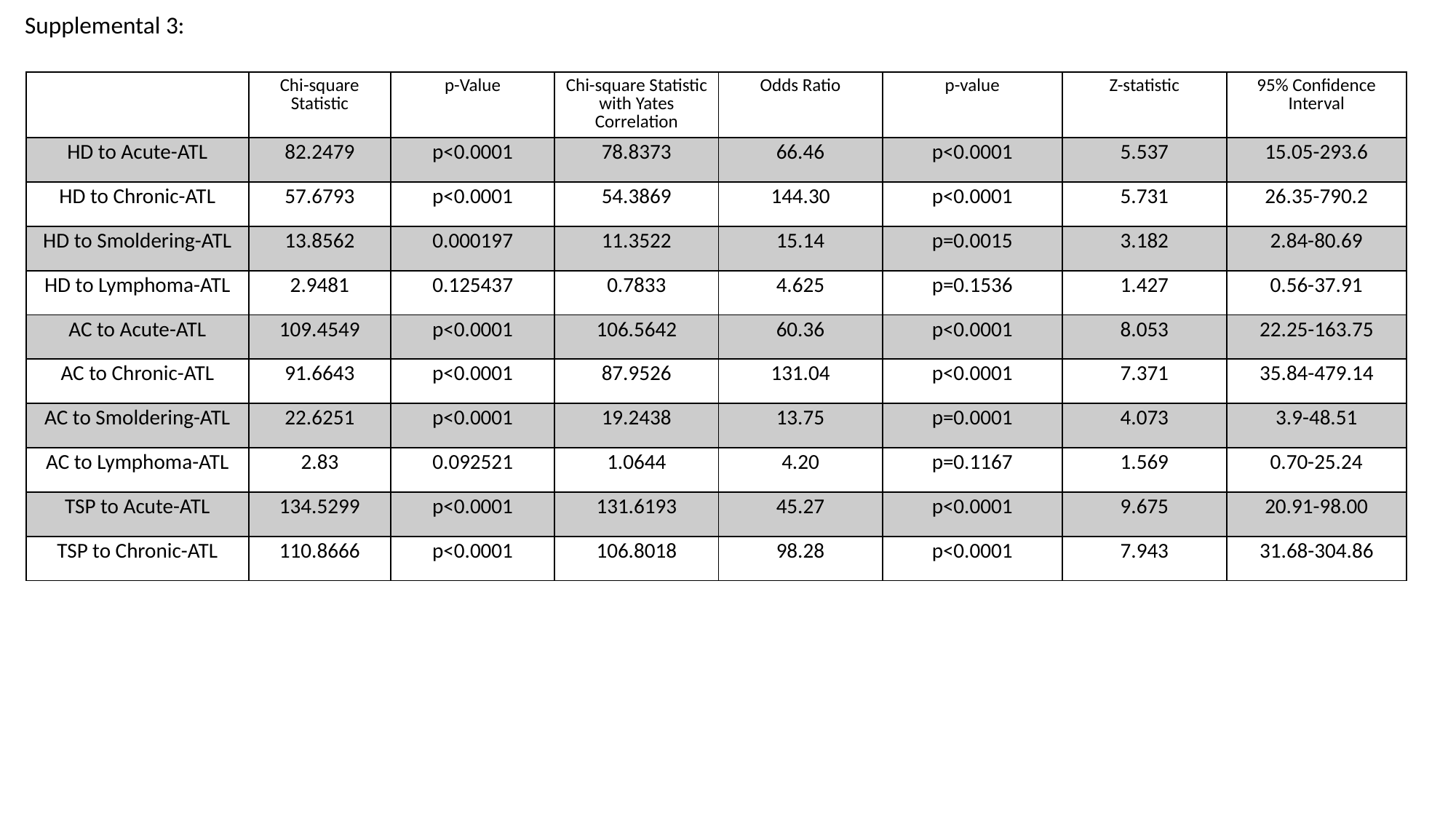

Supplemental 3:
| | Chi-square Statistic | p-Value | Chi-square Statistic with Yates Correlation | Odds Ratio | p-value | Z-statistic | 95% Confidence Interval |
| --- | --- | --- | --- | --- | --- | --- | --- |
| HD to Acute-ATL | 82.2479 | p<0.0001 | 78.8373 | 66.46 | p<0.0001 | 5.537 | 15.05-293.6 |
| HD to Chronic-ATL | 57.6793 | p<0.0001 | 54.3869 | 144.30 | p<0.0001 | 5.731 | 26.35-790.2 |
| HD to Smoldering-ATL | 13.8562 | 0.000197 | 11.3522 | 15.14 | p=0.0015 | 3.182 | 2.84-80.69 |
| HD to Lymphoma-ATL | 2.9481 | 0.125437 | 0.7833 | 4.625 | p=0.1536 | 1.427 | 0.56-37.91 |
| AC to Acute-ATL | 109.4549 | p<0.0001 | 106.5642 | 60.36 | p<0.0001 | 8.053 | 22.25-163.75 |
| AC to Chronic-ATL | 91.6643 | p<0.0001 | 87.9526 | 131.04 | p<0.0001 | 7.371 | 35.84-479.14 |
| AC to Smoldering-ATL | 22.6251 | p<0.0001 | 19.2438 | 13.75 | p=0.0001 | 4.073 | 3.9-48.51 |
| AC to Lymphoma-ATL | 2.83 | 0.092521 | 1.0644 | 4.20 | p=0.1167 | 1.569 | 0.70-25.24 |
| TSP to Acute-ATL | 134.5299 | p<0.0001 | 131.6193 | 45.27 | p<0.0001 | 9.675 | 20.91-98.00 |
| TSP to Chronic-ATL | 110.8666 | p<0.0001 | 106.8018 | 98.28 | p<0.0001 | 7.943 | 31.68-304.86 |

## Slide 4
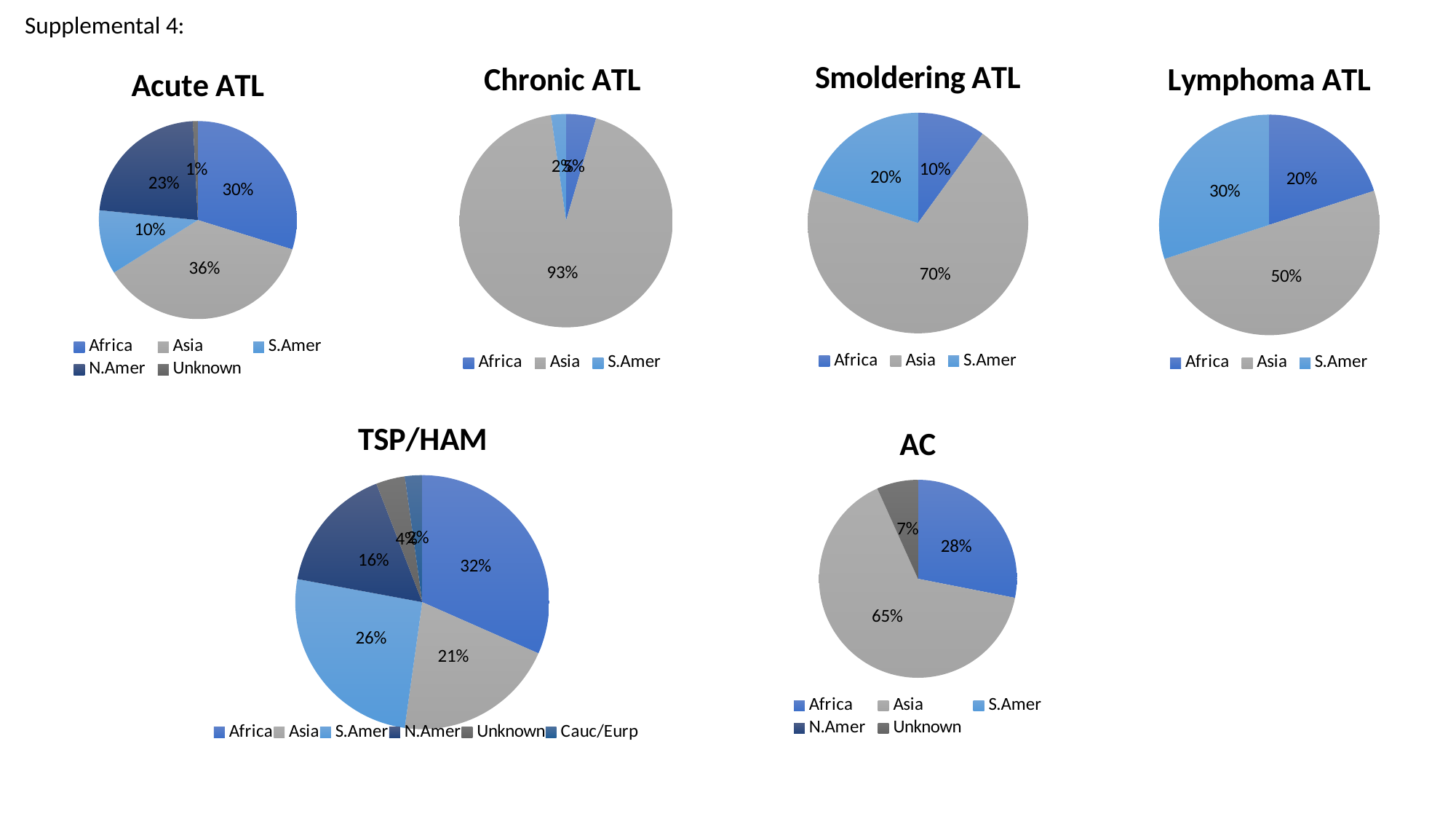

Supplemental 4:
### Chart: Smoldering ATL
| Category | Smoldering |
|---|---|
| Africa | 10.0 |
| Asia | 70.0 |
| S.Amer | 20.0 |
### Chart: Lymphoma ATL
| Category | Lymphoma |
|---|---|
| Africa | 20.0 |
| Asia | 50.0 |
| S.Amer | 30.0 |
### Chart: Chronic ATL
| Category | Chronic |
|---|---|
| Africa | 4.55 |
| Asia | 93.2 |
| S.Amer | 2.27 |
### Chart: Acute ATL
| Category | Acute |
|---|---|
| Africa | 29.8 |
| Asia | 36.3 |
| S.Amer | 10.5 |
| N.Amer | 22.6 |
| Unknown | 0.806 |
### Chart: TSP/HAM
| Category | TSP/HAM |
|---|---|
| Africa | 31.6 |
| Asia | 20.6 |
| S.Amer | 25.7 |
| N.Amer | 16.2 |
| Unknown | 3.68 |
| Cauc/Eurp | 2.2 |
### Chart: AC
| Category | AC |
|---|---|
| Africa | 28.1 |
| Asia | 65.2 |
| S.Amer | 0.0 |
| N.Amer | 0.0 |
| Unknown | 6.74 |

## Slide 5
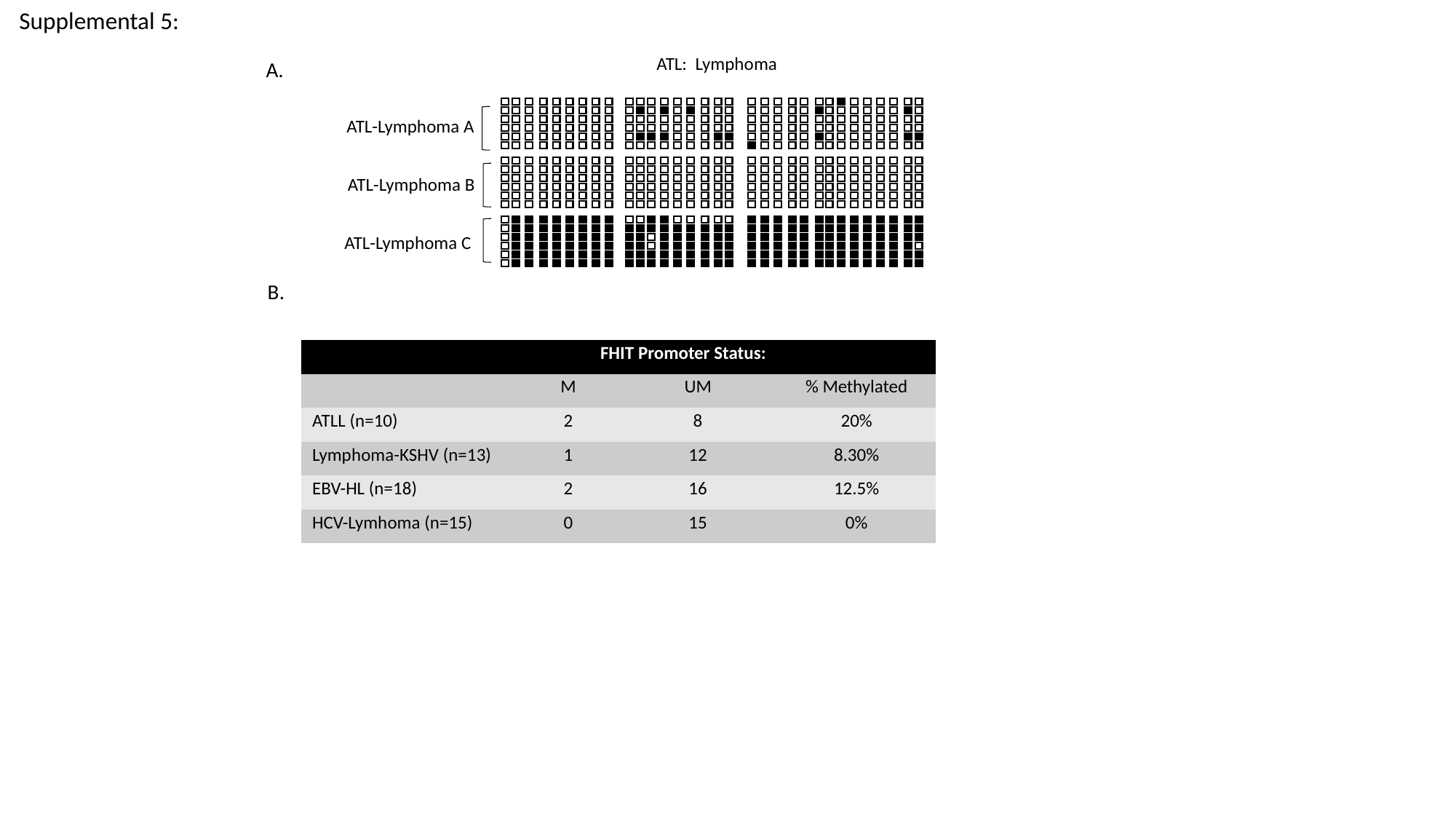

Supplemental 5:
ATL: Lymphoma
A.
ATL-Lymphoma A
ATL-Lymphoma B
ATL-Lymphoma C
B.
| FHIT Promoter Status: | | | |
| --- | --- | --- | --- |
| | M | UM | % Methylated |
| ATLL (n=10) | 2 | 8 | 20% |
| Lymphoma-KSHV (n=13) | 1 | 12 | 8.30% |
| EBV-HL (n=18) | 2 | 16 | 12.5% |
| HCV-Lymhoma (n=15) | 0 | 15 | 0% |

## Slide 6
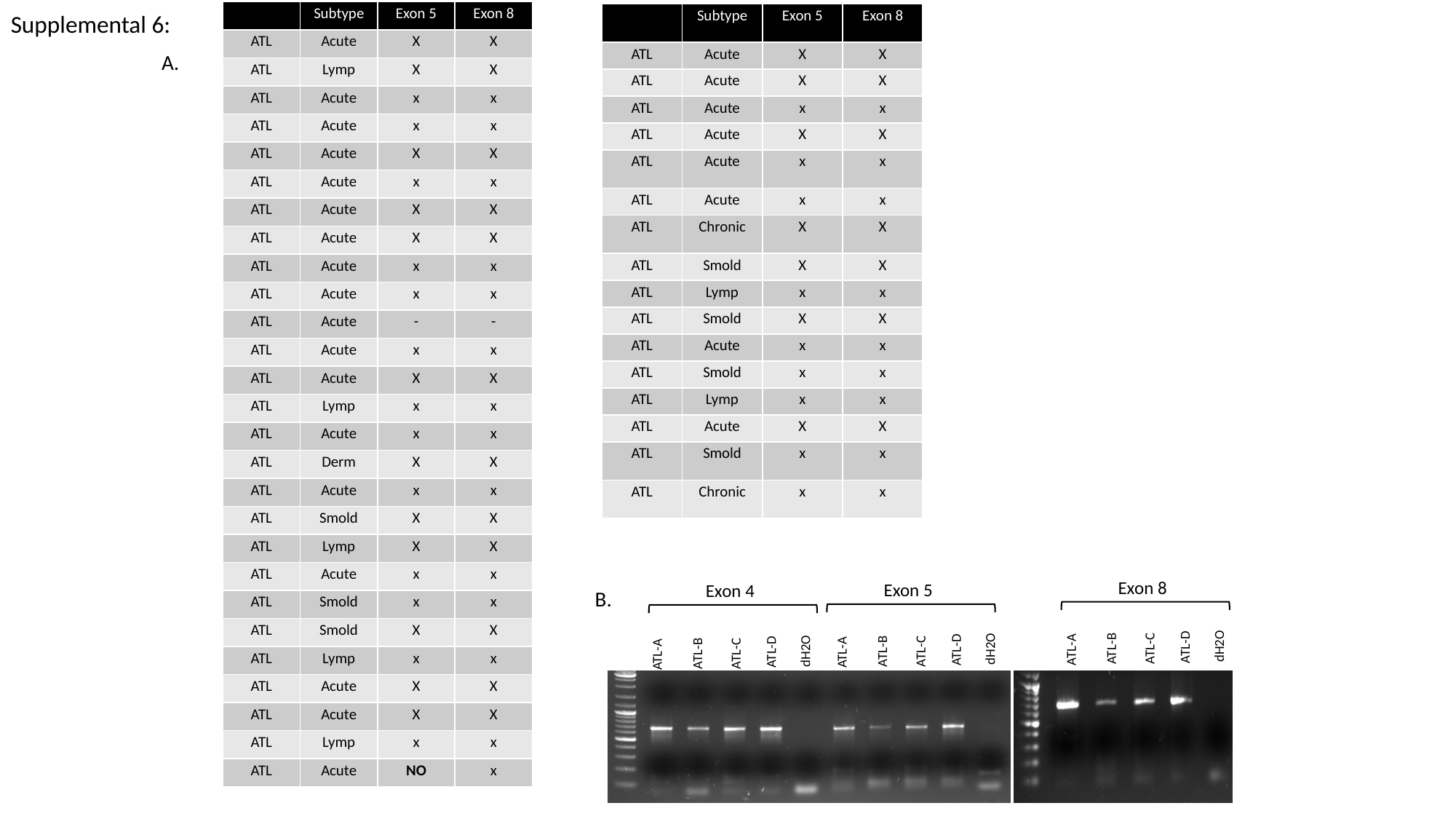

| | Subtype | Exon 5 | Exon 8 |
| --- | --- | --- | --- |
| ATL | Acute | X | X |
| ATL | Lymp | X | X |
| ATL | Acute | x | x |
| ATL | Acute | x | x |
| ATL | Acute | X | X |
| ATL | Acute | x | x |
| ATL | Acute | X | X |
| ATL | Acute | X | X |
| ATL | Acute | x | x |
| ATL | Acute | x | x |
| ATL | Acute | - | - |
| ATL | Acute | x | x |
| ATL | Acute | X | X |
| ATL | Lymp | x | x |
| ATL | Acute | x | x |
| ATL | Derm | X | X |
| ATL | Acute | x | x |
| ATL | Smold | X | X |
| ATL | Lymp | X | X |
| ATL | Acute | x | x |
| ATL | Smold | x | x |
| ATL | Smold | X | X |
| ATL | Lymp | x | x |
| ATL | Acute | X | X |
| ATL | Acute | X | X |
| ATL | Lymp | x | x |
| ATL | Acute | NO | x |
Supplemental 6:
| | Subtype | Exon 5 | Exon 8 |
| --- | --- | --- | --- |
| ATL | Acute | X | X |
| ATL | Acute | X | X |
| ATL | Acute | x | x |
| ATL | Acute | X | X |
| ATL | Acute | x | x |
| ATL | Acute | x | x |
| ATL | Chronic | X | X |
| ATL | Smold | X | X |
| ATL | Lymp | x | x |
| ATL | Smold | X | X |
| ATL | Acute | x | x |
| ATL | Smold | x | x |
| ATL | Lymp | x | x |
| ATL | Acute | X | X |
| ATL | Smold | x | x |
| ATL | Chronic | x | x |
A.
Exon 8
Exon 5
Exon 4
B.
dH2O
ATL-D
ATL-C
ATL-B
dH2O
ATL-A
ATL-D
dH2O
ATL-C
ATL-B
ATL-D
ATL-A
ATL-C
ATL-B
ATL-A
