# Supplementary material for: Germinal epimutation of Fragile Histidine Triad (FHIT) gene is associated with progression to acute and chronic adult T-cell leukemia diseases
Source: Mol Cancer. 2021 Jun 6;20:86. doi: 10.1186/s12943-021-01370-2 (PMC8183032; doi:10.1186/s12943-021-01370-2)
Supplement: Supplementary file 2 — Additional file 2: Supplemental Table 1. [file 12943_2021_1370_MOESM2_ESM.doc]

|  | **Origin:** | **Disease:** | **Sequenced:** | **FHIT Status:** | **PVL:** |
| --- | --- | --- | --- | --- | --- |
| 1 | S.Amer | ATL |  | **M** | NA |
| 2 | S.Amer | ATL |  | **M** | 31.2 |
| 3 | S.Amer | ATL |  | **M** | 77.2 |
| 4 | S.Amer | ATL |  | **UM** | 7.16 |
| 5 | S.Amer | ATL | BGS | **M** | 34.4 |
| 6 | S.Amer | ATL |  | **M** | 53.8 |
| 7 | S.Amer | ATL |  | **M** | 40.51 |
| 8 | S.Amer | ATL |  | **UM** | 7.87 |
| 9 | S.Amer | ATL |  | **M** | 26.6 |
| 10 | Africa | ATL |  | **M** | 701 |
| 11 | Africa | ATL |  | **M** | 1.42 |
| 12 | Africa | ATL |  | **M** | 163 |
| 13 | Africa | ATL |  | **M** | 338 |
| 14 | Africa | ATL |  | **M** | 167 |
| 15 | Africa | ATL |  | **M** | 253 |
| 16 | Africa | ATL |  | **M** | 1887 |
| 17 | N.Amer | ATL |  | **M** | 555 |
| 18 | N.Amer | ATL |  | **M** | 24.2 |
| 19 | N.Amer | ATL | BGS | **M** | 20.0 |
| 20 | UN | ATL |  | **M** | 69.2 |
| 21 | Africa | ATL |  | **M** | 78.4 |
| 22 | Africa | ATL |  | **M** | NA |
| 23 | Africa | ATL |  | **M** | NA |
| 24 | Africa | ATL |  | **M** | 157 |
| 25 | Africa | ATL | BGS | **M** | 8.28 |
| 26 | Africa | ATL | BGS | **M** | 5.16 |
| 27 | Africa | ATL | BGS | **M** | 67.1 |
| 28 | Africa | ATL | BGS | **M** | 5.6 |
| 29 | Africa | ATL | BGS | **M** | 26.96 |
| 30 | Africa | ATL |  | **M** | 48.4 |
| 31 | Africa | ATL |  | **UM** | 263 |
| 32 | Africa | ATL |  | **M** | 15.1 |
| 33 | Africa | ATL |  | **UM** | 0.70 |
| 34 | Africa | ATL | BGS | M | 5.12 |
| 35 | Africa | ATL | BGS | **UM** | 98.5 |
| 36 | Africa | ATL |  | **M** | 31.3 |
| 37 | Africa | ATL |  | **UM** | 0.015 |
| 38 | Africa | ATL |  | **M** | 86.0 |
| 39 | Africa | ATL | BGS | **M** | 73.2 |
| 40 | Africa | ATL |  | **M** | 222 |
| 41 | Africa | ATL |  | **M** | NA |
| 42 | Africa | ATL | BGS | **M** | 195 |
| 43 | Africa | ATL |  | **UM** | 2.39 |
| 44 | Africa | ATL | BGS | **M** | 18.5 |
| 45 | Africa | ATL | BGS | **M** | 153 |
| 46 | Africa | ATL | BGS | **M** | 4.23 |
| 47 | Africa | ATL |  | **M** | 31.0 |
| 48 | N.Amer | ATL |  | **UM** | 58.4 |
| 49 | N.Amer | ATL | BGS | **M** | 50.6 |
| 50 | N.Amer | ATL |  | **UM** | 279 |
| 51 | N.Amer | ATL | BGS | **M** | 144 |
| 52 | N.Amer | ATL |  | **UM** | 63.7 |
| 53 | N.Amer | ATL | BGS | **M** | 41.3 |
| 54 | N.Amer | ATL | BGS | **UM** | 15.0 |
| 55 | Africa | ATL | BGS | **M** | 12.4 |
| 56 | Africa | ATL |  | **M** | 129 |
| 57 | Africa | ATL |  | **M** | 154.5 |
| 58 | Asia | ATL |  | **M** | 51.0 |
| 59 | Asia | ATL | BGS | **M** | 23.2 |
| 60 | Asia | ATL | BGS | **M** | 31.6 |
| 61 | Asia | ATL | BGS | **M** | 105 |
| 62 | Asia | ATL |  | **M** | 38.4 |
| 63 | Asia | ATL |  | **M** | 104 |
| 64 | Asia | ATL |  | **UM** | 57.3 |
| 65 | Asia | ATL |  | **M** | 61.3 |
| 66 | Asia | ATL |  | **UM** | 22.9 |
| 67 | Asia | ATL |  | **M** | 52.7 |
| 68 | Asia | ATL |  | **M** | 103 |
| 69 | Asia | ATL | BGS | **M** | 47.6 |
| 70 | Asia | ATL |  | **M** | 65.4 |
| 71 | Asia | ATL | BGS | **M** | 18.6 |
| 72 | Asia | ATL |  | **M** | 120 |
| 73 | Asia | ATL | BGS | **M** | 39.9 |
| 74 | Asia | ATL |  | **M** | 39.9 |
| 75 | Asia | ATL |  | **M** | 45.1 |
| 76 | Asia | ATL |  | **M** | 53.0 |
| 77 | Asia | ATL |  | **M** | 177 |
| 78 | Asia | ATL |  | **M** | 36.1 |
| 79 | Asia | ATL | BGS | **M** | 33.0 |
| 80 | Asia | ATL | BGS | **M** | 26.2 |
| 81 | Asia | ATL | BGS | **M** | 9.44 |
| 82 | Asia | ATL |  | **UM** | 194 |
| 83 | Asia | ATL | BGS | **M** | 3.51 |
| 84 | Asia | ATL |  | **UM** | 4.50 |
| 85 | Asia | ATL |  | **M** | 222 |
| 86 | Asia | ATL | BGS | **M** | 53.9 |
| 87 | Asia | ATL | BGS | **M** | 71.3 |
| 88 | Asia | ATL |  | **UM** | 0.0008 |
| 89 | Asia | ATL | BGS | **M** | 15.2 |
| 90 | Asia | ATL | BGS | **M** | 87.2 |
| 91 | Asia | ATL | BGS | **M** | 54.7 |
| 92 | Asia | ATL |  | **UM** | 2.08 |
| 93 | Asia | ATL | BGS | **M** | 8.6 |
| 94 | Asia | ATL | BGS | **M** | 112 |
| 95 | Asia | ATL | BGS | **UM** | 122 |
| 96 | Asia | ATL | BGS | **M** | 56 |
| 97 | Asia | ATL |  | **UM** | 365 |
| 98 | Asia | ATL | BGS | **M** | 203 |
| 99 | Asia | ATL | BGS | **M** | 22 |
| 100 | Asia | ATL | BGS | **M** | 66 |
| 101 | Asia | ATL | BGS | **M** | 65 |
| 102 | Asia | ATL | BGS | **M** | 181 |
| 103 | N.Amer | ATL |  | **UM** | 7.93 |
| 104 | N.Amer | ATL |  | **M** | 16.0 |
| 105 | N.Amer | ATL |  | **M** | 20.3 |
| 106 | N.Amer | ATL |  | **M** | 16.4 |
| 107 | N.Amer | ATL | BGS | **M** | 4.4 |
| 108 | N.Amer | ATL |  | **M** | 14.9 |
| 109 | N.Amer | ATL | BGS | **UM** | 3.02 |
| 110 | N.Amer | ATL |  | **M** | 19.4 |
| 111 | N.Amer | ATL |  | **UM** | 5.18 |
| 112 | N.Amer | ATL |  | **UM** | 3.86 |
| 113 | N.Amer | ATL |  | **M** | 8.23 |
| 114 | N.Amer | ATL |  | **UM** | 4.45 |
| 115 | N.Amer | ATL |  | **M** | 14.2 |
| 116 | N.Amer | ATL |  | **M** | 49.4 |
| 117 | N.Amer | ATL |  | **M** | 56.6 |
| 118 | N.Amer | ATL |  | **M** | 16.3 |
| 119 | N.Amer | ATL |  | **UM** | 20.2 |
| 120 | S.Amer | ATL |  | **M** | 12.1 |
| 121 | S.Amer | ATL | BGS | **UM** | 0.54 |
| 122 | S.Amer | ATL |  | **UM** | 0.07 |
| 123 | S.Amer | ATL | BGS | **M** | 33.1 |
| 124 | N.Amer | ATL |  | **M** | NA |
|  |  |  |  |  |  |
|  |  |  |  |  |  |
|  |  |  |  |  |  |
| 1 | Africa | Chronic |  | M | 118 |
| 2 | Asia | Chronic |  | **M** | 75.3 |
| 3 | Asia | Chronic |  | **M** | 37.6 |
| 4 | Asia | Chronic |  | **M** | 55.0 |
| 5 | Asia | Chronic |  | **M** | 8.98 |
| 6 | Asia | Chronic |  | **M** | 30.7 |
| 7 | Asia | Chronic |  | **M** | 21.4 |
| 8 | Asia | Chronic | BGS | **UM** | 15.8 |
| 9 | Asia | Chronic |  | **M** | 37.8 |
| 10 | Asia | Chronic | BGS | **M** | 56.6 |
| 11 | Asia | Chronic | BGS | **M** | 72 |
| 12 | Asia | Chronic | BGS | **M** | 103 |
| 13 | Africa | Chronic | BGS | **M** | 0.003 |
| 14 | S.Amer | Chronic | BGS | **M** | 47.2 |
| 15 | Asia | Chronic |  | **M** | 21.8 |
| 16 | Asia | Chronic | BGS | **M** | 22.52 |
| 17 | Asia | Chronic |  | **M** | 11.75 |
| 18 | Asia | Chronic |  | **M** | 11.14 |
| 19 | Asia | Chronic | BGS | **UM** | 23.13 |
| 20 | Asia | Chronic | BGS | **M** | 22.52 |
| 21 | Asia | Chronic |  | **M** | 11.18 |
| 22 | Asia | Chronic |  | **M** | 6.49 |
| 23 | Asia | Chronic | BGS | **M** | 27.06 |
| 24 | Asia | Chronic | BGS | **UM** | 18.96 |
| 25 | Asia | Chronic | BGS | **M** | 44.5 |
| 26 | Asia | Chronic |  | **UM** | 1.73 |
| 27 | Asia | Chronic |  | **M** | 303 |
| 28 | Asia | Chronic | BGS | **UM** | 15.4 |
| 29 | Asia | Chronic | BGS | **M** | 36.2 |
| 30 | Asia | Chronic |  | **M** | 25.7 |
| 31 | Asia | Chronic | BGS | **M** | 19.2 |
| 32 | Asia | Chronic | BGS | **M** | 33.8 |
| 33 | Asia | Chronic | BGS | **M** | 35.8 |
| 34 | Asia | Chronic | BGS | **M** | 83.6 |
| 35 | Asia | Chronic | BGS | **M** | 35.8 |
| 36 | Asia | Chronic | BGS | **M** | 80.8 |
| 37 | Asia | Chronic | BGS | **M** | 42.8 |
| 38 | Asia | Chronic | BGS | **M** | 31.8 |
| 39 | Asia | Chronic | BGS | **M** | 80.2 |
| 40 | Asia | Chronic | BGS | **M** | 45.9 |
| 41 | Asia | Chronic | BGS | **M** | 13.8 |
| 42 | Asia | Chronic |  | **M** | 120 |
| 43 | Asia | Chronic |  | **M** | 38.2 |
| 44 | Asia | Chronic |  | **M** | 30.9 |
|  |  |  |  |  |  |
|  |  |  |  |  |  |
|  |  |  |  |  |  |
|  |  |  |  |  |  |
| 1 | Africa | Lymphoma | BGS | **M** | 126 |
| 2 | Africa | Lymphoma |  | **UM** | 298 |
| 3 | Asia | Lymphoma |  | **UM** | 1.67 |
| 4 | Asia | Lymphoma | BGS | **M** | 8.05 |
| 5 | Asia | Lymphoma |  | **UM** | 3.95 |
| 6 | Asia | Lymphoma | BGS | **UM** | 0.015 |
| 7 | S.Amer | Lymphoma |  | **UM** | 0.26 |
| 8 | S.Amer | Lymphoma | BGS | **UM** | 2.06 |
| 9 | S.Amer | Lymphoma |  | **UM** | 9.03 |
| 10 | Asia | Lymphoma | BGS | **UM** | 0.24 |
|  |  |  |  |  |  |
|  |  |  |  |  |  |
| 1 | S.Amer | Smoldering | BGS | **M** | 10.8 |
| 2 | S.Amer | Smoldering | BGS | **UM** | 3.91 |
| 3 | S.Amer | Smoldering | BGS | **UM** | 54.4 |
| 4 | S.Amer | Smoldering | BGS | **UM** | 22.2 |
| 5 | Asia | Smoldering | BGS | **M** | 22 |
| 6 | Asia | Smoldering | BGS | **UM** | 30.5 |
| 7 | Asia | Smoldering | BGS | **M** | 22 |
| 8 | Asia | Smoldering | BGS | **M** | 97 |
| 9 | Asia | Smoldering | BGS | **UM** | 23 |
| 10 | Asia | Smoldering | BGS | **M** | 30.5 |
| 11 | Asia | Smoldering | BGS | **M** | 115 |
| 12 | Asia | Smoldering |  | **UM** | 1.6 |
| 13 | Asia | Smoldering |  | **UM** | 1 |
| 14 | Africa | Smoldering |  | **M** | 242 |
| 15 | Asia | Smoldering |  | **UM** | 3.22 |
| 16 | Africa | Smoldering |  | **M** | 109 |
| 17 | Asia | Smoldering | BGS | **UM** | 4.25 |
| 18 | Asia | Smoldering | BGS | **UM** | 8.65 |
| 19 | Asia | Smoldering | BGS | **UM** | 0.8 |
| 20 | Asia | Smoldering | BGS | **M** | NA |
|  |  |  |  |  |  |
| 1 | Asia | Derm.ATL |  | **UM** | 0.00006 |
|  |  |  |  |  |  |
|  |  | **ATL** | **198** | **UM: 51** | **25.8%** |
|  |  |  |  | **M: 147** | **74.2%** |
|  |  |  |  |  |  |
|  |  | **Acute** | **124** | **UM: 27**  **M: 97** | **21.8%**  **78.2%** |
|  |  | **Chronic** | **44** | **UM: 5**  **M: 39** | **11.4%**  **88.6%** |
|  |  | **Smoldering** | **20** | **UM: 11**  **M: 9** | **55%**  **45%** |
|  |  | **Lymphoma** | **10** | **UM: 8**  **M: 2** | **80%**  **20%** |
|  |  | **Dermis** | **1** | **UM: 1**  **M: 0** | **100%** |
|  |  |  |  |  |  |
|  |  |  |  |  |  |
| 1 |  | HD |  | **UM** | 0.035 |
| 2 |  | HD |  | **UM** | 0.018 |
| 3 |  | HD |  | **UM** | 0.151 |
| 4 |  | HD |  | **UM** | 1.60 |
| 5 |  | HD |  | **UM** | 0.49 |
| 6 |  | HD |  | **UM** | 1.32 |
| 7 |  | HD |  | **UM** | 0.70 |
| 8 |  | HD |  | **UM** | 0.32 |
| 9 |  | HD |  | **UM** | 0.31 |
| 10 |  | HD |  | **UM** | 0.47 |
| 11 |  | HD |  | **UM** | 0.99 |
| 12 |  | HD |  | **UM** | 0.033 |
| 13 |  | HD |  | **UM** | 0.70 |
| 14 |  | HD |  | **UM** | 5.65 |
| 15 |  | HD |  | **UM** | 0.11 |
| 16 |  | HD |  | **UM** | 0.04 |
| 17 |  | HD |  | **UM** | 0.21 |
| 18 |  | HD |  | **UM** | 0.092 |
| 19 |  | HD |  | **UM** | 0.016 |
| 20 |  | HD |  | **UM** | 1.05 |
| 21 |  | HD |  | **M** | 0.005 |
| 22 |  | HD |  | **UM** | 0.016 |
| 23 |  | HD | BGS | **UM** | 0.007 |
| 24 |  | HD | BGS | **M** | 0.015 |
| 25 |  | HD |  | **UM** | 0.0007 |
| 26 |  | HD |  | **UM** | 0.348 |
| 27 |  | HD |  | **UM** | 0.165 |
| 28 |  | HD |  | **UM** | 0.079 |
| 29 |  | HD |  | **UM** | 0.14 |
| 30 |  | HD | BGS | **UM** | 0.11 |
| 31 |  | HD | BGS | **UM** | 0.03 |
| 32 |  | HD | BGS | **UM** | 18.9 |
| 33 |  | HD |  | **UM** | 0.03 |
| 34 |  | HD |  | **UM** | 0.012 |
| 35 |  | HD |  | **UM** | 0.067 |
| 36 |  | HD |  | **UM** | 0.06 |
| 37 |  | HD | *Quality Issue* | *NA* | 0.025 |
| 38 |  | HD |  | **UM** | 0.07 |
| 39 |  | HD | BGS | **UM** | 0.02 |
| 40 |  | HD |  | **UM** | NA |
|  |  |  |  |  |  |
|  |  |  |  |  |  |
|  |  | **HD** | **39** | **UM: 37** | **94.9%** |
|  |  |  |  | **M: 2** | **5.13%** |
|  |  |  | **NA** | **1** |  |
|  |  |  |  |  |  |
| 1 | N.Amer | TSP |  | **UM** | 9.13 |
| 2 | N.Amer | TSP | BGS | **UM** | 0.91 |
| 3 | N.Amer | TSP | *Quality Issue* | *NA* | 2.61 |
| 4 | N.Amer | TSP |  | **UM** | 0.23 |
| 5 | N.Amer | TSP |  | **UM** | 0.86 |
| 6 | N.Amer | TSP | BGS | **UM** | 19.1 |
| 7 | N.Amer | TSP | BGS | **UM** | 13.1 |
| 8 | N.Amer | TSP | BGS | **UM** | 151 |
| 9 | N.Amer | TSP | BGS | **M** | 6.04 |
| 10 | Africa | TSP |  | **UM** | 5.61 |
| 11 | Africa | TSP |  | **UM** | 4.52 |
| 12 | Africa | TSP |  | **UM** | 0.68 |
| 13 | Africa | TSP |  | **UM** | 0.01 |
| 14 | Africa | TSP |  | **UM** | 8.45 |
| 15 | Africa | TSP |  | **UM** | 21.5 |
| 16 | Africa | TSP |  | **UM** | 20.5 |
| 17 | Africa | TSP |  | **UM** | 59.3 |
| 18 | Africa | TSP | *Quality Issue* | *NA* | 19.5 |
| 19 | Africa | TSP |  | **UM** | 48.1 |
| 20 | Africa | TSP |  | **UM** | 27.1 |
| 21 | Africa | TSP |  | **UM** | 56.2 |
| 22 | Africa | TSP |  | **UM** | 25.5 |
| 23 | Africa | TSP |  | **UM** | 1.71 |
| 24 | Africa | TSP | BGS | **M** | 224 |
| 25 | Africa | TSP |  | **UM** | 8.87 |
| 26 | Africa | TSP |  | **UM** | 16.0 |
| 27 | Africa | TSP |  | **UM** | 20.7 |
| 28 | Africa | TSP |  | **UM** | 15.5 |
| 29 | Africa | TSP |  | **UM** | 20.5 |
| 30 | Africa | TSP |  | **UM** | 2.41 |
| 31 | Africa | TSP |  | **UM** | 10.6 |
| 32 | Africa | TSP |  | **UM** | 1.87 |
| 33 | Africa | TSP |  | **UM** | 9.9 |
| 34 | Africa | TSP | BGS | **UM** | 41.1 |
| 35 | Africa | TSP |  | **UM** | 25.7 |
| 36 | Africa | TSP |  | **UM** | 6.65 |
| 37 | Africa | TSP |  | **UM** | 0.86 |
| 38 | Africa | TSP |  | **UM** | 10.6 |
| 39 | Africa | TSP |  | **UM** | 49.7 |
| 40 | Africa | TSP |  | **UM** | 0.833 |
| 41 | Africa | TSP |  | **UM** | 0.0 |
| 42 | Africa | TSP |  | **UM** | 12.4 |
| 43 | Africa | TSP |  | **UM** | 7.24 |
| 44 | Africa | TSP |  | **UM** | 5.05 |
| 45 | Africa | TSP |  | **UM** | 1.89 |
| 46 | Africa | TSP |  | **UM** | 4.49 |
| 47 | Africa | TSP | *Quality Issue* | *NA* | 11.3 |
| 48 | Africa | TSP |  | **UM** | 1.07 |
| 49 | Africa | TSP |  | **UM** | NA |
| 50 | Africa | TSP |  | **UM** | NA |
| 51 | Africa | TSP |  | **UM** | NA |
| 52 | Africa | TSP |  | **UM** | 13.2 |
| 53 | Africa | TSP |  | **UM** | 17.2 |
| 54 | S.Amer | TSP |  | **UM** | 3.13 |
| 55 | S.Amer | TSP |  | **UM** | 15.9 |
| 56 | S.Amer | TSP |  | **UM** | 0.02 |
| 57 | S.Amer | TSP |  | **UM** | 3.05 |
| 58 | S.Amer | TSP |  | **UM** | 8.42 |
| 59 | S.Amer | TSP |  | **UM** | NA |
| 60 | S.Amer | TSP | BGS | **M** | NA |
| 61 | S.Amer | TSP |  | **UM** | NA |
| 62 | S.Amer | TSP |  | **UM** | NA |
| 63 | S.Amer | TSP | BGS | **UM** | 3.92 |
| 64 | S.Amer | TSP |  | **UM** | 0.43 |
| 65 | S.Amer | TSP | BGS | **UM** | NA |
| 66 | S.Amer | TSP |  | **UM** | 10.4 |
| 67 | S.Amer | TSP | BGS | **UM** | NA |
| 68 | S.Amer | TSP |  | **UM** | 7.69 |
| 69 | S.Amer | TSP |  | **UM** | 0.01 |
| 70 | S.Amer | TSP |  | **UM** | 1.13 |
| 71 | S.Amer | TSP |  | **UM** | 4.97 |
| 72 | S.Amer | TSP |  | **UM** | NA |
| 73 | S.Amer | TSP |  | **UM** | NA |
| 74 | Asia | TSP | BGS | **UM** | .008 |
| 75 | Asia | TSP |  | **UM** | 1.27 |
| 76 | Asia | TSP | BGS | **M** | 4.3 |
| 77 | Asia | TSP |  | **UM** | 0.26 |
| 78 | Asia | TSP | *Quality Issue* | *NA* | 0.97 |
| 79 | Asia | TSP |  | **UM** | 0.00006 |
| 80 | Asia | TSP |  | **UM** | 1.59 |
| 81 | Asia | TSP |  | **UM** | 2.44 |
| 82 | Asia | TSP | BGS | **UM** | 4.67 |
| 83 | Asia | TSP | BGS | **M** | 28.1 |
| 84 | Asia | TSP |  | **UM** | 0.47 |
| 85 | Asia | TSP | BGS | **UM** | 4.18 |
| 86 | Asia | TSP |  | **UM** | 0.7 |
| 87 | Asia | TSP |  | **UM** | 3.25 |
| 88 | Asia | TSP |  | **UM** | 1.19 |
| 89 | Asia | TSP | BGS | **M** | 1.95 |
| 90 | Asia | TSP |  | **UM** | 3.2 |
| 91 | Asia | TSP |  | **UM** | 0.25 |
| 92 | Asia | TSP |  | **UM** | 3.02 |
| 93 | Asia | TSP |  | **UM** | 1.95 |
| 94 | Asia | TSP | BGS | **UM** | 0.44 |
| 95 | Asia | TSP | BGS | **UM** | 0.88 |
| 96 | Asia | TSP |  | **UM** | 0.02 |
| 97 | Asia | TSP |  | **UM** | 4.79 |
| 98 | Asia | TSP |  | **UM** | 0.30 |
| 99 | Asia | TSP |  | **UM** | 0.4 |
| 100 | Asia | TSP |  | **UM** | 1.82 |
| 101 | N.Amer | TSP |  | **UM** | 8.63 |
| 102 | N.Amer | TSP |  | **UM** | 3.25 |
| 103 | Asia | TSP |  | **UM** | 16.4 |
| 104 | N.Amer | TSP | BGS | **UM** | 7.35 |
| 105 | N.Amer | TSP | BGS | **M** | 13.3 |
| 106 | Asia | TSP | BGS | **UM** | 12.1 |
| 107 | Cauc | TSP |  | **UM** | 5.24 |
| 108 | Cauc | TSP |  | **UM** | 0.76 |
| 109 | N.Amer | TSP |  | **UM** | 3.62 |
| 110 | Cauc | TSP |  | **UM** | 1.51 |
| 111 | N.Amer | TSP |  | **UM** | 5.72 |
| 112 | N.Amer | TSP |  | **UM** | 1.83 |
| 113 | Africa | TSP | BGS | **UM** | 1.66 |
| 114 | N.Amer | TSP |  | **UM** | 4.73 |
| 115 | N.Amer | TSP |  | **UM** | 5.22 |
| 116 | S.Amer | TSP |  | **UM** | 5.74 |
| 117 | N.Amer | TSP |  | **UM** | 1.35 |
| 118 | N.Amer | TSP |  | **UM** | 3.85 |
| 119 | N.Amer | TSP |  | **UM** | 11.5 |
| 120 | N.Amer | TSP |  | **UM** | 11.7 |
| 121 | N.Amer | TSP |  | **UM** | 4.03 |
| 122 | S.Amer | TSP | BGS | **UM** | NA |
| 123 | S.Amer | TSP | BGS | **UM** | NA |
| 124 | S.Amer | TSP | BGS | **UM** | NA |
| 125 | S.Amer | TSP | BGS | **UM** | NA |
| 126 | S.Amer | TSP | BGS | **M** | NA |
| 127 | S.Amer | TSP | BGS | **UM** | NA |
| 128 | S.Amer | TSP | *Quality Issue* | *NA* | NA |
| 129 | S.Amer | TSP | *Quality Issue* | *NA* | NA |
| 130 | S.Amer | TSP | BGS | **UM** | NA |
| 131 | S.Amer | TSP | BGS | **M** | NA |
| 132 | S.Amer | TSP | BGS | **UM** | NA |
| 133 | S.Amer | TSP | BGS | **UM** | NA |
| 134 | S.Amer | TSP | BGS | **UM** | NA |
| 135 | S.Amer | TSP | *Quality Issue* | *NA* | NA |
| 136 | S.Amer | TSP | BGS | **M** | NA |
| 137 | S.Amer | TSP | BGS | **UM** | NA |
| 138 | S.Amer | TSP | *Quality Issue* | *NA* | NA |
| 139 | S.Amer | TSP | BGS | **UM** | NA |
| 140 | UN | TSP | BGS | **UM** | NA |
| 141 | UN | TSP |  | **UM** | NA |
| 142 | UN | TSP |  | **UM** | NA |
| 143 | UN | TSP |  | **UM** | NA |
| 144 | UN | TSP |  | **UM** | NA |
|  |  |  |  |  |  |
|  |  |  |  |  |  |
|  |  |  |  |  |  |
|  |  | **TSP** | **136** | **UM: 126** |  |
|  |  |  |  | **M: 10** |  |
|  |  |  | **NA** | **8** |  |
|  |  |  |  |  |  |
|  |  |  |  |  |  |
|  |  |  |  |  |  |
| 1 | Africa | AS |  | **UM** | 1.70 |
| 2 | Africa | AS |  | **UM** | 0.55 |
| 3 | Africa | AS |  | **UM** | 0.016 |
| 4 | Africa | AS |  | **UM** | 7.98 |
| 5 | Africa | AS |  | **UM** | 3.64 |
| 6 | Africa | AS |  | **UM** | 1.81 |
| 7 | Africa | AS |  | **UM** | 0.205 |
| 8 | Africa | AS |  | **UM** | .00009 |
| 9 | Africa | AS |  | **UM** | 0.028 |
| 10 | Africa | AS | BGS | **UM** | 3.65 |
| 11 | Africa | AS | *Quality Issue* | *NA* | *0.007* |
| 12 | Africa | AS |  | **UM** | 1.85 |
| 13 | Africa | AS |  | **UM** | 0.46 |
| 14 | Africa | AS |  | **UM** | 0.0015 |
| 15 | UN | AS |  | **UM** | 99.1 |
| 16 | UN | AS |  | **UM** | 50.6 |
| 17 | UN | AS |  | **UM** | 166 |
| 18 | UN | AS |  | **UM** | 88.1 |
| 19 | UN | AS | *Quality Issue* | *NA* | 344 |
| 20 | UN | AS |  | **UM** | 135 |
| 21 | Africa | AS |  | **UM** | 15.6 |
| 22 | Africa | AS |  | **UM** | 7.21 |
| 23 | Africa | AS |  | **UM** | 0.72 |
| 24 | Africa | AS |  | **UM** | 6.73 |
| 25 | 65.2 | AS |  | **UM** | 8.20 |
| 26 | Asia | AS |  | **UM** | 1.66 |
| 27 | Asia | AS |  | **UM** | 0.76 |
| 28 | Asia | AS |  | **M** | 18.2 |
| 29 | Asia | AS |  | **UM** | 3.99 |
| 30 | Asia | AS |  | **UM** | 0.004 |
| 31 | Asia | AS |  | **UM** | 0.0022 |
| 32 | Asia | AS |  | **UM** | 1.01 |
| 33 | Asia | AS |  | **UM** | 7.17 |
| 34 | Asia | AS |  | **UM** | 0.71 |
| 35 | Asia | AS |  | **UM** | 0.23 |
| 36 | Asia | AS |  | **UM** | 1.25 |
| 37 | Asia | AS |  | **UM** | 1.2 |
| 38 | Asia | AS |  | **UM** | 3.48 |
| 39 | Asia | AS |  | **UM** | 14.67 |
| 40 | Asia | AS |  | **UM** | 0.003 |
| 41 | Asia | AS |  | **M** | 5.92 |
| 42 | Asia | AS |  | **UM** | 3.83 |
| 43 | Asia | AS |  | **UM** | 1.57 |
| 44 | Asia | AS |  | **UM** | 6.80 |
| 45 | Asia | AS |  | **UM** | 5.86 |
| 46 | Asia | AS |  | **UM** | 0.85 |
| 47 | Asia | AS |  | **UM** | 0.21 |
| 48 | Asia | AS |  | **UM** | 0.11 |
| 49 | Asia | AS |  | **UM** | 0.41 |
| 50 | Asia | AS |  | **UM** | 1.47 |
| 51 | Asia | AS |  | **UM** | 2.73 |
| 52 | Asia | AS |  | **UM** | 0.48 |
| 53 | Asia | AS |  | **UM** | 0.52 |
| 54 | Asia | AS |  | **UM** | 0.003 |
| 55 | Asia | AS |  | **M** | 8.26 |
| 56 | Asia | AS |  | **UM** | 0.181 |
| 57 | Asia | AS |  | **UM** | 1.97 |
| 58 | Asia | AS |  | **UM** | 0.26 |
| 59 | Asia | AS |  | **UM** | 0.01 |
| 60 | Asia | AS |  | **UM** | 0.34 |
| 61 | Asia | AS |  | **UM** | 3.48 |
| 62 | Asia | AS |  | **UM** | 0.298 |
| 63 | Asia | AS |  | **UM** | 0.0025 |
| 64 | Asia | AS |  | **UM** | 3.99 |
| 65 | Asia | AS |  | **UM** | 0.923 |
| 66 | Asia | AS |  | **UM** | 0.967 |
| 67 | Asia | AS |  | **UM** | 0.031 |
| 68 | Asia | AS |  | **UM** | 0.076 |
| 69 | Asia | AS |  | **UM** | 0.167 |
| 70 | Asia | AS |  | **UM** | 2.17 |
| 71 | Asia | AS |  | **UM** | 0.426 |
| 72 | Asia | AS |  | **UM** | 2.75 |
| 73 | Asia | AS |  | **UM** | 2.28 |
| 74 | Asia | AS |  | **UM** | 1.49 |
| 75 | Africa | AS | BGS | **UM** | NA |
| 76 | Africa | AS | BGS | **UM** | NA |
| 77 | Africa | AS | BGS | **UM** | NA |
| 78 | Africa | AS | BGS | **UM** | NA |
| 79 | Africa | AS | BGS | **UM** | NA |
| 80 | Africa | AS | BGS | **UM** | NA |
| 81 | Africa | AS | BGS | **UM** | NA |
| 82 | Asia | AS |  | **UM** | NA |
| 83 | Asia | AS | *Quality Issue* | *NA* | NA |
| 84 | Asia | AS |  | **UM** | NA |
| 85 | Asia | AS |  | **UM** | NA |
| 86 | Asia | AS |  | **UM** | NA |
| 87 | Asia | AS | *Quality Issue* | *NA* | NA |
| 88 | Asia | AS |  | **UM** | NA |
| 89 | Asia | AS |  | **UM** | NA |
| 90 | Asia | AS | *Quality Issue* | *NA* | NA |
| 91 | Asia | AS |  | **UM** | NA |
| 92 | Asia | AS |  | **M** | NA |
| 93 | Asia | AS |  | **M** | NA |
| 94 | Asia | AS |  | **UM** | NA |
|  |  |  |  |  |  |
|  |  |  |  |  |  |
|  |  |  |  |  |  |
|  |  | **AC** | **89** | **UM: 84** | **94.4%** |
|  |  |  |  | **M: 5** | **5.62 %** |
|  |  |  | **NA** | **5** |  |
|  |  |  |  |  |  |
